# Optimising plasma clozapine levels to improve treatment response: an individual patient data meta-analysis and receiver operating characteristic curve analysis

**DOI:** 10.1192/bjp.2023.27

**Published:** 2023-06

**Authors:** Korinne Northwood, E. Pearson, U. Arnautovska, S. Kisely, M. Pawar, M. Sharma, K. Vitangcol, E. Wagner, N. Warren, Dan Siskind

**Affiliations:** Metro South Addiction and Mental Health Service, Metro South Health, Australia and Faculty of Medicine, University of Queensland, Australia; College of Medicine and Public Health, Flinders University, Australia; Metro South Addiction and Mental Health Service, Metro South Health, Australia; Department of Mental Health, Monash Health, Australia; Faculty of Medicine, University of Queensland, Australia; Department of Psychiatry and Psychotherapy, University Hospital, LMU Munich, Germany

**Keywords:** Clozapine, optimisation, ROC curve, individual patient data meta-analysis, therapeutic drug monitoring

## Abstract

**Background:**

Although clozapine is the most efficacious medication for treatment-refractory schizophrenia, not all patients will have an adequate response. Optimising clozapine dose using therapeutic drug monitoring could therefore maximise response.

**Aims:**

Using individual patient data, we undertook a receiver operating characteristic (ROC) curve analysis to determine an optimal therapeutic range for clozapine levels to guide clinical practice.

**Method:**

We conducted a systematic review of PubMed, PsycINFO and Embase for studies that provided individual participant level data on clozapine levels and response. These data were analysed using ROC curves to determine the prediction performance of plasma clozapine levels for treatment response.

**Results:**

We included data on 294 individual participants from nine studies. ROC analysis yielded an area under the curve of 0.612. The clozapine level at the point of optimal diagnostic benefit was 372 ng/mL; at this level, the response sensitivity was 57.3%, and specificity 65.7%. The interquartile range for treatment response was 223–558 ng/mL. There was no improvement in ROC performance with mixed models including patient gender, age or length of trial. Clozapine dose and clozapine concentration to dose ratio did not provide significantly meaningful prediction of response to clozapine.

**Conclusions:**

Clozapine dose should be optimised based on clozapine therapeutic levels. We found that a range between 250 and 550 ng/mL could be recommended, while noting that a level of >350 ng/mL is the most optimal for response. Although some patients may not respond without clozapine levels >550 ng/mL, the benefits should be weighed against the increased risk of adverse drug reactions.

## Background

One in three people with schizophrenia have treatment-resistant schizophrenia, with ongoing psychotic symptoms despite two adequate trials of at least two antipsychotic agents.^1^ Clozapine is the most effective antipsychotic for reducing positive symptoms^2^ and hospital admissions.^3^ However, only 40% of people with treatment-resistant schizophrenia will have an adequate response to clozapine.^4^

In clinical practice, it is important to clarify whether people with clozapine-resistant schizophrenia have true clozapine resistance, or ‘pseudo-resistance’ as a result of inadequate clozapine plasma levels to achieve a response. Existing studies on the optimal therapeutic range for clozapine levels have been limited by small sample sizes or analyses based on prior assumptions of estimated therapeutic range.^5^

## Aims

Receiver operating characteristic (ROC) curve analysis can evaluate drug efficacy by classifying responses based on a continuous variable,^6^ and as such, can provide information about the optimal range for a treatment response above a certain sensitivity threshold. Using individual patient data from previously published studies, we undertook a ROC curve analysis to more comprehensively determine an optimal therapeutic range for clozapine levels to guide clinical practice.

## Method

This systematic review was conducted according to the Preferred Reporting Items for Systematic Reviews and Meta-Analyses for Individual Patient Data systematic reviews (PRISMA-IPD) guidelines.^7^ The protocol, submitted prior to the search, is available through PROSPERO, an international database of prospectively registered systematic review protocols, registration number CRD42021242181. This study extends the original protocol by including analysis of individual patient data.

### Searches

A systematic search of PubMed, PsycINFO and Embase was undertaken from database inception up to 23 August 2022 using the search terms (Clozapin* OR Clozaril OR Zaponex OR Denzapin* OR Clopine OR Norclozapine OR Desmethylclozapine) AND (level OR levels OR concentration OR concentrations OR ratio OR ratios) AND (blood OR serum OR plasma) (Supplementary Table 1 available at https://doi.org/10.1192/bjp.2023.27 – PubMed Search Terms). Articles were dually screened by two authors, first at the title and abstract level, then at full-text level. Key researchers in the field of clozapine and treatment-resistant schizophrenia were contacted regarding unpublished data and individual patient data. Data extraction was conducted by one author and validated by another author.

### Inclusion and exclusion criteria

Cohort studies, case series, case–control studies and randomised and non-randomised controlled trials were included, but single case reports were excluded. We did not exclude studies based on language. To be meaningful for ROC analysis, studies needed to provide data on individual patient's clozapine levels at end-point, and either:

- whether or not participants achieved a threshold for response of 20% reduction in Positive and Negative Syndrome Scale (PANSS) or Brief Psychiatric Rating Scale (BPRS) as per criteria set out by Kane et al,^8^ or
- provide data on participant's total psychosis symptoms as rated by the PANSS or BPRS at time of clozapine initiation and end-point so that response could be calculated.

Information on study duration, clozapine level, clozapine dose, study setting, country of study, diagnostic tool, antipsychotic comedications, definition of treatment resistance, and where available, data on age, gender, illness duration and duration of trial (in weeks) of participants was also extracted.

### Study quality

Study quality was rated using a modified Newcastle–Ottawa Scale^9^ (Supplementary Tables 2 and 3), with a maximum score of 5. A score of ≥3 points for a study was assigned as high quality with a low risk of bias. The domains of assessment were: sample representativeness, sample size, clarity of definition of response, ascertainment of clozapine levels, quality of reporting.

### Analyses

Analysis was carried out within the R programming environment.^10^ ROC curve analysis was performed using R package *ROCit*. As data were not normally distributed, a non-parametric model was applied using the function *rocit* with *method = ‘non’.* Area under the curve (AUC) was calculated as the aggregate measure of classification performance, with an AUC value of >0.5 representing greater than chance likelihood of prediction response using clozapine level. Precision estimates of >55% were set as relative cut-offs for both specificity and sensitivity. Response rate and number needed to treat (NNT) were calculated for patients within and above the interquartile range indicated by ROC analysis, using the patients below the therapeutic range as the ‘control’ grouping.

Package *cutpointr* was employed for exploration of Youden Index and clozapine level cut-points. The Youden Index is a measure of ‘informedness’ that allows generalisation of the dichotomous outcome for the test being examined; in this case, it is a measure of how informative a particular clozapine plasma value may be for predicting response. Youden Index values range from 0 to 1, with values closer to 1 indicating a more sensitive and specific test. For this analysis, both sensitivity and specificity of the test are weighted equally. Analyses were also conducted on daily clozapine dose, and clozapine concentration to dose ratio (levels in ng/mL divided by dose in mg per day).

Further mixed models, incorporating combinations models of patient gender, time on treatment and response, were analysed using the *glm* function within base R package *stats,* followed by creation of a ROC object using function *rocit* as described above. Graphing was performed using package *ggplot2*.

## Results

In total, 7694 studies were identified at title and abstract level, after removal of duplicates. Following screening at the title and abstract level, 270 studies were reviewed at full-text level, with ten studies meeting inclusion criteria, including eight where individual patient data had been published. We contacted authors of the two potentially relevant studies where data was unpublished, inviting them to provide their individual patient data. One author provided data^11^ and one did not. The final number of studies for inclusion was nine (see Supplementary Table 4 for included studies, Supplementary Figure 1 for the PRISMA flow diagram, Supplementary Table 5 for the PRISMA-IPD checklist and Supplementary Table 8: excluded studies.)

### Study characteristics

The nine included studies provided data on 294 individual participants.^11–19^ Most studies came from the USA (*n* = 6) with one each from Australia, Italy and Saudi Arabia.

The mean duration of the included studies was 10.4 weeks, with an s.d. of 8.2 and a range of 4 to 24 weeks. There was no statistically significant difference between length of trial for responders and non-responders. Mean age of participants was 32.6 years (s.d. = 9.08), and 69.5% of participants were male. Age and gender did not differ between responders and non-responders. The mean daily clozapine dose was 421 mg (s.d. = 160 mg), with a mean clozapine level of 427 ng/mL (s.d. = 297 ng/mL) (see Supplementary Table 6 for demographics by response).

All participants were either on no other antipsychotic medication at the start of the trial, or had their previous antipsychotic medication cross-tapered during clozapine titration. Titration protocols varied between studies (Supplementary Table 4: included studies). Treatment response criteria were reported to be ≥20% reduction in PANSS or BPRS in all studies, with eight studies including an additional criterion of end-point BPRS of ≥34 or a Clinical Global Impression score of ≥3.

All studies reported that clozapine testing was done on plasma trough levels. One study^15^ contained clozapine values in interval measures of 50 ng/mL, and the remainder all had continuous values. All studies were of prospective design.

Study quality was good overall, with all studies rated as being of high quality (Supplementary Table 3: risk of bias). Over half (*n* = 7) of the studies had <50 participants, and three did not provide sufficient data to compare the gender and age of participants between responder and non-responder groups.

### ROC analysis

**Fig. 1:**
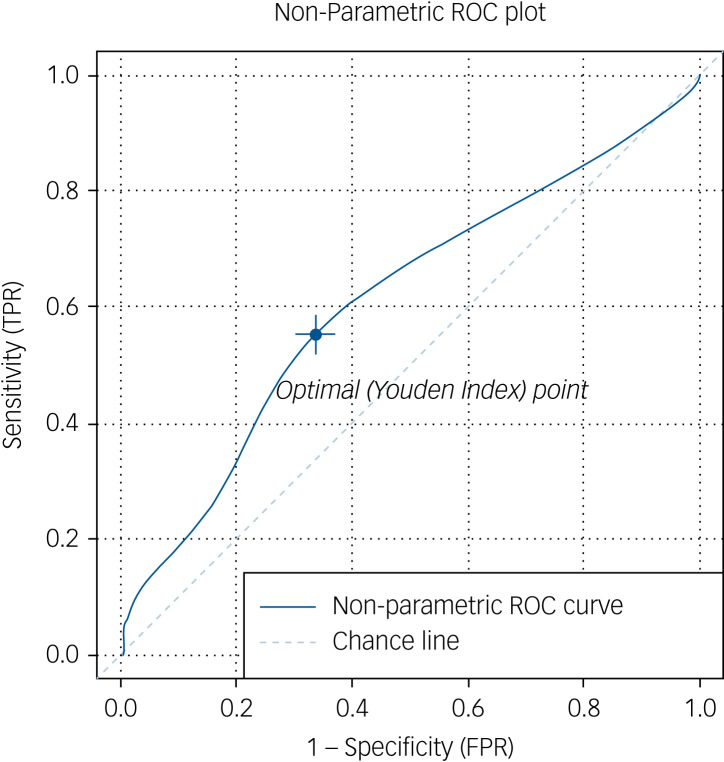
Receiver operating characteristic (ROC) analysis of clozapine treatment response. Non-parametric ROC analysis demonstrates that therapeutic level (solid line) is a better classifier of clozapine treatment response then chance (dotted line), with an area under the curve of 0.612 (95% CI 0.54**–**0.68). The Youden Index, indicating point of optimal response prediction was found at the clozapine level of 372 ng/mL, with a response specificity of 57.3% and sensitivity of 65.7%. FPR, false positive rate; TPR, true positive rate.

Non-parametric ROC curve analysis demonstrated an AUC of 0.612 (95% CI 0.54–0.68), indicating that clozapine levels provide a greater than chance predictor of treatment response (Fig. 1). The Youden Index, a global measure of response ‘informedness’, indicating the optimal point along the ROC curve for response prediction, was calculated at a clozapine level of 372 ng/mL, with a response specificity of 57.3% and sensitivity of 65.7% at this level.

**Fig. 2:**
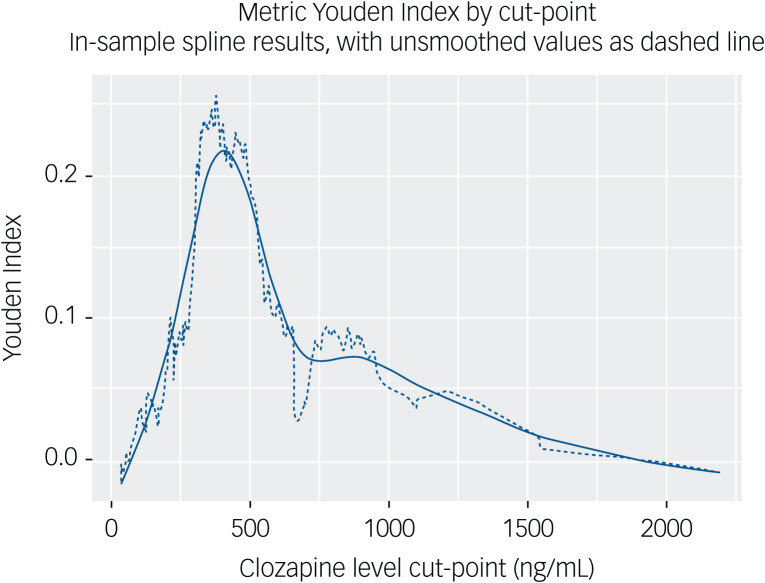
Metric Youden Index by clozapine level cut-point. Youden Index integrates specificity and sensitivity to determine the point of highest ‘informedness’ for prediction of response by clozapine level, with Loess smoothing applied as the solid line over unsmoothed values. Youden Index peaks at clozapine levels between 350 and 500 ng/mL, tapering to zero from levels of >800 ng/mL.

To further explore the optimal cut-point for determining treatment response, we calculated the metric Youden Index across all cut-points in the data-set (Fig. 2), with Loess smoothing to account for the ‘stepped’ clozapine levels provided from one study. This analysis confirmed the optimal response point at a clozapine level of 372 ng/mL, with an interquartile range of 223–558 ng/mL. It also revealed a more narrow ‘optimal window’ of response with Youden value of >0.2 for clozapine levels between 308 ng/mL and 481 ng/mL.

**Fig. 3:**
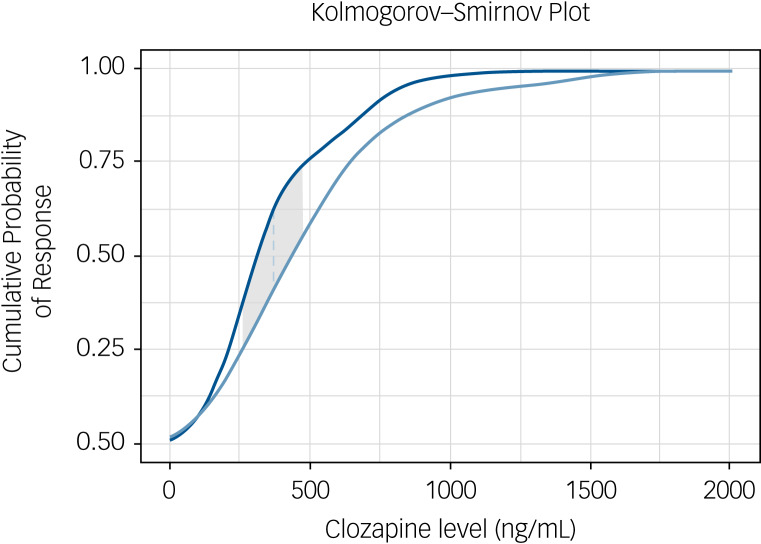
Two-sample Kolmogorov–Smirnov plot for cumulative distribution of positive and negative response values. Two-sample Kolmogorov–Smirnov test demonstrates that the non-responder patient population (light-blue line) and the responder patient population (dark-blue line) represent two distinct but similarly distributed populations (*P* = 0.21). The responder population demonstrates higher cumulative probability of response, diverging from the non-responder population at a clozapine level of ~250 ng/mL. The dashed blue line indicates the point of the Kolmogorov–Smirnov statistic, which is the widest divergence of the two populations, at 376.3 ng/mL, with grey shaded area representing the 95% confidence interval for prediction of response.

Two-sample Kolmogorov–Smirnov testing for probability response revealed that clozapine level distribution was statistically similar across both responder and non-responder groups (*P* = 0.21), indicating that these groups can be treated as being drawn from the same population, and having similar distribution of clozapine levels. The Kolmogorov–Smirnov statistic, calculated at the maximum difference between the cumulative distribution functions for positive and negative responses, was determined at a clozapine level of 376.3 ng/mL, comparable with that determined using the Youden value (Fig. 3).

In order to assist clinical decision-making, we calculated the response rates and NNT for patients within and above the interquartile range of 223–558 ng/mL indicated by the ROC analysis. Within our recommended range, the response rate was 53.2%, with an NNT of 5.5. Above this range the NNT was much higher at 17.2, with a response rate of 40.8%.

Mixed models exploring whether patient gender, age or length of trial would have an impact on response did not demonstrate any improvement in response prediction above the base model (Supplementary Table 7: sensitivity analyses).

Non-parametric ROC curve analysis on clozapine dose demonstrated that dose alone was unhelpful for predicting clozapine response, with an AUC of 0.44 (95% CI 0.32–0.56). There was no meaningful Youden Index for this analysis. Clozapine concentration to dose ratio was also not as useful as clozapine level alone for predicting treatment benefit, with an AUC of 0.56 (95% CI 0.44–0.68) (Supplementary Figure 2: non-parametric ROC curve for clozapine dose and concentration-dose ratio).

Publication bias could not be calculated as there were fewer than ten studies in the analysis.

## Discussion

### Main findings

Our work has used collective data sources and ROC curve analysis to determine optimal clozapine levels for which treatment response can be expected. We demonstrated the usefulness of clozapine levels for response determination and identified a level of ~370 ng/mL as the optimal index for response, with an interquartile range of 223–558 ng/mL.

### Comparison with findings from other studies

This therapeutic range for clozapine levels aligns with ranges discussed in existing clozapine consensus guidelines^20^ and previous studies.^5,21^ Ensuring clozapine levels are in the therapeutic range is recommended as a first step for patients with ongoing psychotic symptoms to ensure that pseudo-resistance is excluded prior to consideration of adding augmentation treatments to clozapine.^20^ Although the evidence for augmentation treatments for clozapine resistance is limited, electroconvulsive therapy,^22,23^ second-generation antipsychotics^22,23^ and cognitive–behavioural therapy for psychosis^24^ have the best evidence.

Higher clozapine levels have been associated with higher levels of adverse drug reactions (ADRs), notably triglycerides, heart rate and all-cause ADRs.^25^ Clozapine levels over 1000 ng/mL have been associated with higher rates of seizures.^26^ Given the increased risks of ADRs with higher clozapine levels, any decision for clozapine dosage for clozapine levels >550 ng/mL must weigh any potential reduction in psychotic symptoms against the risk of ADRs.

In our analysis, all included studies used a consistent, previously recognised, definition of response, >20% reduction in PANSS or BPRS.^8^ In addition, all clozapine testing was reported to have been done at trough levels.

When analyses were conducted on clozapine dose, and clozapine concentration to dose ratio, neither were able to provide meaningful Youden indices, suggesting that these tests are not useful in predicting response to clozapine. This is in keeping with previous studies that found clozapine dose and concentration to dose ratio were unhelpful clinically for predicting response.^5^

### Limitations

Mixed modelling which included patient gender, age or length of clozapine trial, did not result in any improvement in response prediction above that of clozapine levels alone. We note that mixed modelling was performed on a smaller sample as only six studies provided information on gender and age, and that this may benefit from further analysis with a larger sample in future. Additionally, our work did not consider the impact of co-prescribed augmentation strategies such as a second antipsychotic or a mood stabiliser agent, as this data was not available. It would be useful to explore a ROC curve model that incorporates this extra information to determine if this might alter the limits of the therapeutic range, or the optimal level for response. Further, included studies did not have individual patient data on ADRs, preventing a meaningful risk–benefit analysis of ADRs and response at different clozapine levels.

As with all systematic reviews, there is a potential bias with inability to access unpublished data. By convention, ten studies are needed before publication bias analysis can be performed, as we had only nine studies, we were unable to perform bias analysis. There are some potential limitations with the individual patient data, including lack of reported information on clozapine testing techniques, such as trough and steady state levels, and laboratory analytical methods. Given these studies were conducted before the development of more recent consensus guidelines on clozapine titration,^27^ titration protocols varied between studies.

There may also be unquantifiable bias of interrater reliability for PANSS/BPRS between studies, and bias because of a time lag between clozapine level and response rating being made. Study duration ranged from 4 to 24 weeks. As such, it is possible that some included studies may not have been of an adequate duration to confirm whether participants would have achieved a response to clozapine.

We did not have information on patient ethnicity, smoking or non-psychotropic cytochrome p450 affecting medications, which are all factors known to have an impact on clozapine metabolism. Our primary outcome was clozapine level and therapeutic response; however, we undertook subanalysis on clozapine dose ratio, which takes into account clozapine metabolism; this was not found to be a useful predictor of response.

One further limitation of our work relates to the problem of minimally important change, which is defined as the smallest change in outcome score that can indicate a meaningful change in patient outcomes. Methodologically, when performing ROC analysis, it is customary to value sensitivity and specificity equally in order to determine cut-point thresholds.^28^ Future analysis allowing consideration of multiple cut-points for percentage change in the PANSS or BPRS would be useful if this data were to become available.^29^ Further the translation of these measures into a binary variable may also have resulted in a loss of meaningful information about treatment response. Analysis of continuous response data may be more beneficial in the determination of optimal cut-points.^30^

### Implications

This work has demonstrated a robust mathematical model for determining appropriate reference ranges for therapeutic drug monitoring of clozapine in treatment-refractory schizophrenia. Based on our model, we recommend a therapeutic range of 250–550 ng/mL, with the most optimal benefits seen above 350 ng/mL. Although some patients will respond at lower clozapine levels and therefore will not need higher doses, patients who fail to respond at the lower limit should be uptitrated to ≥350 ng/mL to ensure an adequate trial. Although some patients may not respond without clozapine levels >550 ng/mL, the benefits should be weighed against the increased risk of ADRs.

## Data Availability

The data that support the findings of this study are available from the corresponding author upon reasonable request.

## Supplementary material

For supplementary material accompanying this paper visit https://doi.org/10.1192/bjp.2023.27. click here to view supplementary material
