# Supplementary material for: Optimising plasma clozapine levels to improve treatment response: an individual patient data meta-analysis and receiver operating characteristic curve analysis
Source: Br J Psychiatry. 2023 Jun;222(6):241–5. doi: 10.1192/bjp.2023.27 (PMC10201335; doi:10.1192/bjp.2023.27)
Supplement: Supplementary file 1 [file S0007125023000272sup001.docx]

Appendix - Supplementary Figures and Tables

[Supplementary Figure 1 – PRISMA IPD Flow Diagram 2](#_Toc127868148)

[Supplementary Figure 2 – Non-parametric ROC Curves for clozapine dose and concentration-dose ratio 3](#_Toc127868149)

[Supplementary Table 1 – PubMed Search Terms 4](#_Toc127868150)

[Supplementary Table 2: Modified Newcastle-Ottawa Scoring Guide 5](#_Toc127868151)

[Supplementary Table 3: Risk of Bias of Included Studies 6](#_Toc127868152)

[Supplementary Table 4: Included Studies 7](#_Toc127868153)

[Supplementary Table 5: PRISMA-IPD Checklist 11](#_Toc127868154)

[Supplementary Table 6 – Demographics by Response 16](#_Toc127868155)

[Supplementary Table 7: Sensitivity Analyses 17](#_Toc127868156)

[Supplementary Table 8: Table of Excluded Studies 18](#_Toc127868157)

# Supplementary Figure 1 – PRISMA IPD Flow Diagram

**PRISMA IPD Flow Diagram**

Articles identified from Databases *:

PubMed (n = 1843)

EMBASE (n = 7299)

PsycInfo (n = 1047)

Number of additional studies identified through other sources including contact with researchers (n = 1 )

Identification

Number of studies after duplicates removed

N = 7694

Reports excluded at full text level:

Inappropriate study type (n = 46)

Incorrect patient population (n = 3)

No usable data (n = 193)

Data not accessible (n = 19)

Number of studies screened for eligibility

N = 270

Number of studies for which IPD were sought

N =10

Number of eligible Studies for which IPD were not sought

N = 6 (Publication date greater than 20 years ago)
Reasons for not seeking IPD should be reported

Number of studies for which IPD were provided n = 9

Number of participants for whom data were provided

N =294

Number participants for whom no data were provided N = 0

Number of studies for which IPD were not provided N =1 (no response from authors)

Number of participants n - 131

Reasons for not providing IPD should be stated

**IPD (report for each main outcome)**

Number of studies included in analysis n = 9

Number of participants included in analysis n = 294

Number participants excluded (give reasons) n = 0

Participants for whom no data were provided (n= )

The PRISMA IPD flow diagram

© Reproduced with permission of the PRISMA IPD Group, which encourages sharing and reuse for non commercial purposes

Available data

Obtaining data

Eligibility

Screening

Analysed data

# Supplementary Figure 2 – Non-parametric ROC Curves for clozapine dose and concentration-dose ratio

Non-parametric ROC curves for A. Clozapine dose and response, and B. clozapine concentration-dose ratio and response. Area under the curve (AUC) for both analyses was less robust than for clozapine level alone, where dose AUC 0.44 (95% C.I. 0.35 – 0.56), ratio AUC 0.56 (95% C.I. 0.44 – 0.68) and level alone AUC 0.61 (95% 0.54 – 0.68). Note that for clozapine dose, there was no meaningful Youden Index identified.

# Supplementary Table 1 – PubMed Search Terms

| Area | PubMed Search Terms |
| --- | --- |
| Clozapine | clozapin* OR clozaril OR zaponex OR denzapin* OR clopine OR norclozapine OR Desmethylclozapine |
| AND | |
| Levels | level OR levels OR concentration OR concentrations OR ratio OR ratios |
| AND | |
| Blood | blood OR serum OR plasma |

# Supplementary Table 2: Modified Newcastle-Ottawa Scoring Guide

| Domain | Score | Criteria |
| --- | --- | --- |
| (**1) Representativeness of the sample** | 1 | Participants on clozapine are similar to the broader population of people on clozapine in terms of age, sex, age of onset of psychotic illness, treatment refractory illness, medication adherence |
|  | 0 | Participants on clozapine are not similar to the broader population of people on clozapine in terms of age, sex, age of onset of psychotic illness, treatment refractory illness, medication adherence |
| **(2) Sample size:** | 1 | Total sample size of included cohort was greater than 50 participants |
|  | 0 | Total sample size of included cohort was less than 50 participants |
| **(3) Clarity of Definition of Response** | 1 | Studies provided clear definition of response, as defined as a change in BPRS or PANSS of at least 20% between clozapine commencement and endpoint |
|  | 0 | Studies did not provide a clear definition of response, as defined as a change in BPRS or PANSS of at least 20% between clozapine commencement and endpoint |
| **(4) Ascertainment of CLZ plasma levels** | 1 | Clozapine plasma levels used in analysis drawn in steady-state under clozapine doses reported as stable |
|  | 0 | Clozapine plasma levels not drawn in steady-state conditions |
| **(5) Quality of reporting** | 1 | Reported descriptive statistics to describe the population including age and sex of individual participants. |
|  | 0 | Descriptive statistics were not reported or were incomplete |

# Supplementary Table 3: Risk of Bias of Included Studies

| Author/year | 1. Representativeness of the sample | 2. Sample size | 3. Clarity of definition of response | 4. Ascertainment of CLZ plasma levels | 5. Quality of descriptive statistics reporting | Total |
| --- | --- | --- | --- | --- | --- | --- |
| Chong, 1997 | 0 | 0 | 1 | 1 | 1 | 3 |
| Hasegawa, 1993 | 1 | 1 | 1 | 1 | 1 | 5 |
| Hussein, 1999 | 1 | 0 | 1 | 1 | 1 | 4 |
| Kronig 1995 | 1 | 0 | 1 | 1 | 1 | 4 |
| Perry, 1991 | 1 | 0 | 1 | 1 | 1 | 4 |
| Pickar, 1992 | 1 | 0 | 1 | 1 | 1 | 4 |
| Potkin 1994 | 1 | 1 | 1 | 1 | 1 | 5 |
| Siskind, 2021 | 1 | 0 | 1 | 1 | 1 | 4 |
| Spina, 2000 | 1 | 0 | 1 | 1 | 1 | 4 |

# Supplementary Table 4: Included Studies

| Author (year) | Country | Setting | Data Collection | Diagnosis | Diagnostic tool | Number of participants (total (male)) meeting criteria in response / non-response group* | Mean age (SD) in response / non-response groups* | Illness duration in years (SD) in response / non-response group* | Study duration | Antipsychotic co-medications | Non-response criteria | Participants. met treatment resistance classification** | Clozapine titration method |
| --- | --- | --- | --- | --- | --- | --- | --- | --- | --- | --- | --- | --- | --- |
| Chong, 1997 | Singapore | Inpatient | Prospective | Schizophrenia | DSM-III-R | 8 (3) / 6 (1) | 31.6 (4.3) / 34.5 (4.7) | 15.4 (4.8) | 12 weeks | None | >20% reduction in BPRS score and either a CGI-severity score ≤3 or BPRS score ≤35) | Yes | To clinical response vs side effects |
| Hasegawa, 1993 | US | Inpatient and outpatient | Prospective | Schizophrenia | DSM-III-R | 30 / 29 | 33.4 (10.2) / 36.4 (11.0) | Not stated | 24 weeks | None | >20% reduction in BPRS score | Yes | Initial dose 25mg, increased by 25 – 50mg every few days to median dose of 400mg, with clinical adjustment thereafter if necessary |
| Hussein, 1999 | Saudi Arabia | Unknown | Prospective | Schizophrenia | Not stated | 11 (8) / 15 (1) | 33.0 (6.3) / 34.1 (11.1) | Not stated | 2-41 months | None | >20% reduction in PANSS score | Yes | Twice daily dosing in 21 patients, single daily dose in 5 patients |
| Kronig, 1995 | US | Inpatient | Prospective | Schizophrenia or schizoaffective disorder | DSM-III | 15 / 22 | 27.9 (6.2) | 8.7 (5.7) | 6 weeks | None | >20% reduction in total BPRS score and either a CGI-severity score ≤3 or BPRS-A score ≤35) | Yes | Titrated as tolerated, with target dose of 500mg/day at day 14 |
| Perry, 1991 | US | Inpatient | Prospective | Schizophrenia | DSM-III-R | 11 (8) / 18 (12) | 34.2 (7.5) / 32.6 (8.1) | 13.6 (5.9) / 13.3 (6.4) | 4 weeks | None | >20% reduction in BPRS and a BPRS score ≤34) | Yes | Titrated as tolerated to clinical effect |
| Pickar, 1992 | US | Inpatient | Prospective | Schizophrenia or schizoaffective disorder | DSM-III-R | 8 (5) / 13 (8) | 31.8 (6.8) / 28.0 (5.4) | 10.8 (8.1) / 11.7 (5.4) | 15 weeks | None | >20% reduction in total BPRS score | Yes | Starting dose 25mg, increased by 25 – 50mg / day for 14 days, with clinical adjustment thereafter if necessary |
| Potkin, 1994 | US | Inpatient | Prospective | Schizophrenia | DSM-III-R | 15 / 35 | Not stated | Not stated | 12 weeks | None | >20% reduction in total BPRS score and either a CGI-severity score ≤3 or BPRS-A score ≤35) | Yes | Standardised titration to 400mg/day over 16 days, then randomly assigned to either 400mg/day or 800mg/day doses. Higher dose group was slowly up-titrated |
| Siskind, 2021 | Australia | Inpatient and outpatient | Prospective | Schizophrenia or schizoaffective disorder | DSM-V | 3 (2) / 9 (8) | 30.0 (12.1) / 35.9 (12.9) | Not stated | 24 weeks | Cross taper from prior antipsychotic | >20% reduction in BPRS score | Yes | Standardised titration for 14days, with clinical adjustment thereafter if necessary |
| Spina, 2000 | Italy | Inpatient and outpatient | Prospective | Schizophrenia | DSM-IV | 18 (15) / 27 (20) | 37.7 (8.8) / 38.4 (11.5) | Not stated | 12 weeks | None | >20% reduction in BPRS score and a BPRS score ≤35) | Yes | Starting dose of 25mg / day, increased by 25 – 50mg / day until median dose of 300mg / day, with clinical adjustment thereafter if necessary |

CCMD = Chinese Classification of Mental Disorders the second edition of the diagnostic criteria for schizophrenia

* Data for entire cohort provided where disaggregated data not available

** TRS criteria adapted from Kane et al 1988: two 6 week trials of different antipsychotics with a chlorpromazine equivalent >600 mg/day

# Supplementary Table 5: PRISMA-IPD Checklist

| **PRISMA-IPD**  **Section/topic** | **Item No** | **Checklist item** | **Reported on page** |
| --- | --- | --- | --- |
| **Title** | | | |
| Title | 1 | Identify the report as a systematic review and meta-analysis of individual participant data. | 1 |
| **Abstract** | | | |
| Structured summary | 2 | Provide a structured summary including as applicable: | 2 |
|  |  | **Background**: state research question and main objectives, with information on participants, interventions, comparators and outcomes. |  |
|  |  | **Methods**: report eligibility criteria; data sources including dates of last bibliographic search or elicitation, noting that IPD were sought; methods of assessing risk of bias. |  |
|  |  | **Results**: provide number and type of studies and participants identified and number (%) obtained; summary effect estimates for main outcomes (benefits and harms) with confidence intervals and measures of statistical heterogeneity. Describe the direction and size of summary effects in terms meaningful to those who would put findings into practice. |  |
|  |  | **Discussion:** state main strengths and limitations of the evidence, general interpretation of the results and any important implications. |  |
|  |  | **Other:** report primary funding source, registration number and registry name for the systematic review and IPD meta-analysis. |  |
| **Introduction** | | | |
| Rationale | 3 | Describe the rationale for the review in the context of what is already known. | 5 |
| Objectives | 4 | Provide an explicit statement of the questions being addressed with reference, as applicable, to participants, interventions, comparisons, outcomes and study design (PICOS). Include any hypotheses that relate to particular types of participant-level subgroups. | 5 |
| **Methods** | | | |
| Protocol and registration | 5 | Indicate if a protocol exists and where it can be accessed. If available, provide registration information including registration number and registry name. Provide publication details, if applicable. | 6 |
| Eligibility criteria | 6 | Specify inclusion and exclusion criteria including those relating to participants, interventions, comparisons, outcomes, study design and characteristics (e.g. years when conducted, required minimum follow-up). Note whether these were applied at the study or individual level i.e. whether eligible participants were included (and ineligible participants excluded) from a study that included a wider population than specified by the review inclusion criteria. The rationale for criteria should be stated. | 6 |
| Identifying studies - information sources | 7 | Describe all methods of identifying published and unpublished studies including, as applicable: which bibliographic databases were searched with dates of coverage; details of any hand searching including of conference proceedings; use of study registers and agency or company databases; contact with the original research team and experts in the field; open adverts and surveys. Give the date of last search or elicitation. | 6 |
| Identifying studies - search | 8 | Present the full electronic search strategy for at least one database, including any limits used, such that it could be repeated. | 6 |
| Study selection processes | 9 | State the process for determining which studies were eligible for inclusion. | 7 |
| Data collection processes | 10 | Describe how IPD were requested, collected and managed, including any processes for querying and confirming data with investigators. If IPD were not sought from any eligible study, the reason for this should be stated (for each such study). | 6 |
|  |  | If applicable, describe how any studies for which IPD were not available were dealt with. This should include whether, how and what aggregate data were sought or extracted from study reports and publications (such as extracting data independently in duplicate) and any processes for obtaining and confirming these data with investigators. |  |
| Data items | 11 | Describe how the information and variables to be collected were chosen. List and define all study level and participant level data that were sought, including baseline and follow-up information. If applicable, describe methods of standardising or translating variables within the IPD datasets to ensure common scales or measurements across studies. | 7 |
| IPD integrity | A1 | Describe what aspects of IPD were subject to data checking (such as sequence generation, data consistency and completeness, baseline imbalance) and how this was done. | 7 |
| Risk of bias assessment in individual studies. | 12 | Describe methods used to assess risk of bias in the individual studies and whether this was applied separately for each outcome. If applicable, describe how findings of IPD checking were used to inform the assessment. Report if and how risk of bias assessment was used in any data synthesis. | 7 |
| Specification of outcomes and effect measures | 13 | State all treatment comparisons of interests. State all outcomes addressed and define them in detail. State whether they were pre-specified for the review and, if applicable, whether they were primary/main or secondary/additional outcomes. Give the principal measures of effect (such as risk ratio, hazard ratio, difference in means) used for each outcome. | 7 |
| Synthesis methods | 14 | Describe the meta-analysis methods used to synthesise IPD. Specify any statistical methods and models used. Issues should include (but are not restricted to):   - Use of a one-stage or two-stage approach. - How effect estimates were generated separately within each study and combined across studies (where applicable). - Specification of one-stage models (where applicable) including how clustering of patients within studies was accounted for. - Use of fixed or random effects models and any other model assumptions, such as proportional hazards. - How (summary) survival curves were generated (where applicable). - Methods for quantifying statistical heterogeneity (such as I^2^ and τ^2^). - How studies providing IPD and not providing IPD were analysed together (where applicable). - How missing data within the IPD were dealt with (where applicable). | 7 & 8 |
| Exploration of variation in effects | A2 | If applicable, describe any methods used to explore variation in effects by study or participant level characteristics (such as estimation of interactions between effect and covariates). State all participant-level characteristics that were analysed as potential effect modifiers, and whether these were pre-specified. |  |
| Risk of bias across studies | 15 | Specify any assessment of risk of bias relating to the accumulated body of evidence, including any pertaining to not obtaining IPD for particular studies, outcomes or other variables. | NA |
| Additional analyses | 16 | Describe methods of any additional analyses, including sensitivity analyses. State which of these were pre-specified. | 8 |
| **Results** | | | |
| Study selection and IPD obtained | 17 | Give numbers of studies screened, assessed for eligibility, and included in the systematic review with reasons for exclusions at each stage. Indicate the number of studies and participants for which IPD were sought and for which IPD were obtained. For those studies where IPD were not available, give the numbers of studies and participants for which aggregate data were available. Report reasons for non-availability of IPD. Include a flow diagram. | 9 |
| Study characteristics | 18 | For each study, present information on key study and participant characteristics (such as description of interventions, numbers of participants, demographic data, unavailability of outcomes, funding source, and if applicable duration of follow-up). Provide (main) citations for each study. Where applicable, also report similar study characteristics for any studies not providing IPD. | 9 |
| IPD integrity | A3 | Report any important issues identified in checking IPD or state that there were none. | none |
| Risk of bias within studies | 19 | Present data on risk of bias assessments. If applicable, describe whether data checking led to the up-weighting or down-weighting of these assessments. Consider how any potential bias impacts on the robustness of meta-analysis conclusions. | 10 |
| Results of individual studies | 20 | For each comparison and for each main outcome (benefit or harm), for each individual study report the number of eligible participants for which data were obtained and show simple summary data for each intervention group (including, where applicable, the number of events), effect estimates and confidence intervals. These may be tabulated or included on a forest plot. | 10 |
| Results of syntheses | 21 | Present summary effects for each meta-analysis undertaken, including confidence intervals and measures of statistical heterogeneity. State whether the analysis was pre-specified, and report the numbers of studies and participants and, where applicable, the number of events on which it is based. | 10 & 11 |
|  |  | When exploring variation in effects due to patient or study characteristics, present summary interaction estimates for each characteristic examined, including confidence intervals and measures of statistical heterogeneity. State whether the analysis was pre-specified. State whether any interaction is consistent across trials. |  |
|  |  | Provide a description of the direction and size of effect in terms meaningful to those who would put findings into practice. |  |
| Risk of bias across studies | 22 | Present results of any assessment of risk of bias relating to the accumulated body of evidence, including any pertaining to the availability and representativeness of available studies, outcomes or other variables. | 12 |
| Additional analyses | 23 | Give results of any additional analyses (e.g. sensitivity analyses). If applicable, this should also include any analyses that incorporate aggregate data for studies that do not have IPD. If applicable, summarise the main meta-analysis results following the inclusion or exclusion of studies for which IPD were not available. | 11 |
| **Discussion** | | | |
| Summary of evidence | 24 | Summarise the main findings, including the strength of evidence for each main outcome. | 13 |
| Strengths and limitations | 25 | Discuss any important strengths and limitations of the evidence including the benefits of access to IPD and any limitations arising from IPD that were not available. | 14 |
| Conclusions | 26 | Provide a general interpretation of the findings in the context of other evidence. | 13 |
| Implications | A4 | Consider relevance to key groups (such as policy makers, service providers and service users). Consider implications for future research. | 15 |
| **Funding** | | | |
| Funding | 27 | Describe sources of funding and other support (such as supply of IPD), and the role in the systematic review of those providing such support. | 4 |

© Reproduced with permission of the PRISMA IPD Group, which encourages sharing and reuse for non-commercial purposes

# Supplementary Table 6 – Demographics by Response

|  | **No Response (N=169)** | **Response (N=125)** | **Total (N=294)** | **p value** |
| --- | --- | --- | --- | --- |
| **Length of trial (weeks)** |  |  |  | 0.326 |
| Mean (SD) | 10.0 (8.0) | 11.0 (8.4) | 10.4 (8.2) |  |
| Range | 4.0 - 24.0 | 4.0 - 24.0 | 4.0 - 24.0 |  |
| **Clozapine level (ng/mL)** |  |  |  | **< 0.001** |
| Mean (SD) | 376.3 (259.2) | 496.1 (331.8) | 427.3 (297.7) |  |
| Range | 37.2 - 2167.3 | 35.7 - 1925.9 | 35.7 - 2167.3 |  |
| **Age (years)** |  |  |  | 0.97 |
| Number where data present | 113 | 86 | 199 |  |
| Mean (SD) | 32.7 (10.4) | 32.6 (7.1) | 32.6 (9.1) |  |
| Range | 19.0 - 61.0 | 21.0 - 48.0 | 19.0 - 61.0 |  |
| **Sex** |  |  |  | 0.161 |
| Number where data present | 113 | 86 | 199 |  |
| Male | 42 (75.0%) | 24 (61.5%) | 66 (69.5%) |  |
| Female | 14 (25.0%) | 15 (38.5%) | 29 (30.5%) |  |
| **Clozapine dose (mg)** |  |  |  | 0.153 |
| Number where data present | 113 | 86 | 199 |  |
| Mean (SD) | 440.9 (175. 5) | 393.8 (130.9) | 421.38 (159. 7) |  |
| Range | 100.0 - 900.0 | 110.0 - 800.0 | 100.0 - 900.0 |  |
| **Clozapine concentration-dose ratio** |  |  |  | 0.138 |
| Number where data present | 113 | 86 | 199 |  |
| Mean (SD) | 1.08 (0.69) | 1.36 (1.12) | 1.19 (0.90) |  |
| Range | 0.16 - 2.85 | 0.26 - 5.54 | 0.16 - 5.54 |  |

# Supplementary Table 7: Sensitivity Analyses

Mixed modelling of response prediction was performed before non-parametric ROC analysis. No mixed model explored demonstrated superior response prediction to the base model of clozapine level only; AUC=Area Under the Curve, C.I. = Confidence Interval.

| Model | AUC (95% C.I.) |
| --- | --- |
| Base (clozapine level only) | 0.612 (0.54 – 0.68) |
| Base + sex | 0.567 (0.44 – 0.68) |
| Base + age | 0.511 (0.39 – 0.63) |
| Base + length of trial | 0.612 (0.55 – 0.68) |
| Base + sex + age | 0.567 (0.44 – 0.68) |
| Base + sex + age + length of trial | 0.584 (0.46 – 0.70) |

# Supplementary Table 8: Table of Excluded Studies

| **Reference** | **Reason for Exclusion** |
| --- | --- |
| (Ackenheil et al., 1976) | No usable data |
| (Adejumo et al., 2021) | Inappropriate study type |
| (Adler et al., 2002) | No usable data |
| (Aissa et al., 2022) | No usable data |
| (Akamine et al., 2017) | No usable data |
| (Albers and Ozdemir, 2004) | No usable data |
| (Alderman, 2004) | Inappropriate study type |
| (Ally and Ally, 2015) | Inappropriate study type |
| (Ambasta et al., 2015) | No usable data |
| (Ambasta et al., 2015) | Inappropriate study type |
| (Arango, 2019) | No usable data |
| (Asenjo Lobos et al., 2010) | Inappropriate study type |
| (Askari et al., 2017) | No usable data |
| (Askari et al., 2017) | No usable data |
| (Assion et al., 2008) | No usable data |
| (Atmaca et al., 2003) | No usable data |
| (Aviles-Gonzalez et al., 2013) | No usable data |
| (Axelsson, 1998) | Data not accessible |
| (Aymard et al., 1997) | Data not accessible |
| (Aymard et al., 1999) | No usable data |
| (Bablenis et al., 1989) | Inappropriate study type |
| (Baker and White, 2004) | No usable data |
| (Balant-Gorgia et al., 1993) | Inappropriate study type |
| (Baldelli et al., 2020) | No usable data |
| (Baldessarini and Frankenburg, 1994) | No usable data |
| (Barnes et al., 2017) | No usable data |
| (Barnes, 2011) | Inappropriate study type |
| (Bartels, 1992) | No usable data |
| (Baumann, 2008) | Inappropriate study type |
| (Bechelli et al., 1994) | Data not accessible |
| (Bell et al., 1998) | Inappropriate study type |
| (Bennett and Keck, 1996) | No usable data |
| (Bennett et al., 2008) | No usable data |
| (Benvenuti et al., 2012) | Data not accessible |
| (Bhattacharya et al., 2021) | No usable data |
| (Bondolfi et al., 1998) | No usable data |
| (Bookholt and Bogers, 2014) | Inappropriate study type |
| (Bowskill et al., 2012) | No usable data |
| (Braga et al., 2009) | Data not accessible |
| (Brau et al., 1978) | No usable data |
| (Brusov et al., 2010) | No usable data |
| (Buckley et al., 1997) | No usable data |
| (Bustillo et al., 2018) | No usable data |
| (Carceller-Sindreu et al., 2014) | No usable data |
| (Carulli et al., 2016) | No usable data |
| (Carulli et al., 2016) | Inappropriate study type |
| (Castberg et al., 2017) | No usable data |
| (Catalan et al., 2011) | No usable data |
| (Catalan et al., 2012) | No usable data |
| (Centorrino et al., 1994) | No usable data |
| (Ceskova, 1995) | Inappropriate study type |
| (Chang et al., 1997) | Data not accessible |
| (Chang et al., 2008) | No usable data |
| (Chang et al., 2012) | No usable data |
| (Charfi, 2013) | No usable data |
| (Chong and Remington, 1998) | No usable data |
| (Chung and Remington, 2005) | Inappropriate study type |
| (Chung et al., 2019a) | No usable data |
| (Chung et al., 2019b) | No usable data |
| (Citrome and Volavka, 2002) | Inappropriate study type |
| (Costa-Dookhan et al., 2020) | Inappropriate study type |
| (Costa-Dookhan et al., 2021) | No usable data |
| (Couchman et al., 2010) | No usable data |
| (Couchman et al., 2012) | No usable data |
| (Couchman et al., 2015) | No usable data |
| (Dal Santo et al., 2020) | No usable data |
| (de Leon et al., 2020a) | Inappropriate study type |
| (de Leon et al., 2020b) | Inappropriate study type |
| (de Leon et al., 2020c) | Inappropriate study type |
| (de Lorenzo et al., 2009) | No usable data |
| (de Lorenzo et al., 2009) | No usable data |
| (De Luca et al., 2019a) | Data not accessible |
| (De Luca et al., 2019b) | Data not accessible |
| (de Oliveira Marques et al., 2017) | No usable data |
| (de Oliveira Marques et al., 2017) | No usable data |
| (Dervaux and Laqueille, 2007) | No usable data |
| (Dettling et al., 2000) | No usable data |
| (Diaz et al., 2018) | No usable data |
| (Dobrinas et al., 2011) | No usable data |
| (Dumortier et al., 1998) | No usable data |
| (Dziedzicka-Wasylewska et al., 2008) | No usable data |
| (Englisch and Zink, 2008) | Inappropriate study type |
| (Esslinger et al., 2010) | No usable data |
| (Fabrazzo et al., 1996) | No usable data |
| (Fabrazzo et al., 2002) | No usable data |
| (Fleischhaker et al., 1998) | No usable data |
| (Frazier et al., 2003) | No usable data |
| (Gaertner et al., 2001) | No usable data |
| (Gaertner et al., 2001) | No usable data |
| (Golden and Honigfeld, 2008) | No usable data |
| (Goldsmith et al., 2017) | No usable data |
| (Gonzalez-Esquivel et al., 2011) | No usable data |
| (Grabski and Kasparek, 2017) | No usable data |
| (Greenwood-Smith et al., 2003) | Inappropriate study type |
| (Grilli-Tissot and Louza, 2014) | Inappropriate study type |
| (Grillo et al., 2007) | No usable data |
| (Grunder et al., 2006) | No usable data |
| (Grundmann et al., 2014) | Inappropriate study type |
| (Guitton et al., 1998a) | No usable data |
| (Guitton et al., 1998b) | No usable data |
| (Gunduz-Bruce et al., 2013) | No usable data |
| (Hantulik et al., 2015) | No usable data |
| (Haring et al., 1990) | No usable data |
| (Haring et al., 1989a) | No usable data |
| (Haring et al., 1989b) | No usable data |
| (Harjaningsih et al., 2021) | No usable data |
| (Hart et al., 2021) | No usable data |
| (Havaki-Kontaxaki et al., 2006) | No usable data |
| (Hermida et al., 2008) | No usable data |
| (Hoimark et al., 2020) | No usable data |
| (Hotham et al., 2014) | No usable data |
| (Huang et al., 2016) | No usable data |
| (Iglesias Garcia et al., 2017) | No usable data |
| (Ignjatovic Ristic et al., 2018) | No usable data |
| (Jann et al., 1997) | No usable data |
| (Jiang et al., 2013) | No usable data |
| (Jiang et al., 2016) | No usable data |
| (Jin, 2014) | No usable data |
| (Jin, 2014) | No usable data |
| (John and Kecanovic, 2021) | Inappropriate study type |
| (Jonsdottir et al., 2010) | No usable data |
| (Jonsson et al., 2019) | No usable data |
| (Kaladjian et al., 1999) | No usable data |
| (Kalter et al., 2020) | No usable data |
| (Kaufmann et al., 2005) | Data not accessible |
| (Keller et al., 2014) | No usable data |
| (Kelly et al., 2011) | Inappropriate study type |
| (Kelly et al., 2020) | No usable data |
| (Keshavarzi et al., 2020) | No usable data |
| (Khan and Preskorn, 2005) | Inappropriate study type |
| (Kikuchi et al., 2015) | No usable data |
| (Kim et al., 2019) | No usable data |
| (Klampfl et al., 2011) | No usable data |
| (Kocar et al., 2018) | Inappropriate study type |
| (Kola Oyewumi et al., 2002) | No usable data |
| (Koole et al., 2019) | No usable data |
| (Krivoy et al., 2021) | No usable data |
| (Kurkar et al., 2015) | No usable data |
| (Kurz et al., 1998) | No usable data |
| (Lammers et al., 1999) | No usable data |
| (Lane et al., 1999) | No usable data |
| (Lane et al., 2001) | No usable data |
| (Langree et al., 2013) | No usable data |
| (Lattuada et al., 2004) | No usable data |
| (Lee et al., 2009) | No usable data |
| (Lee et al., 2012) | Inappropriate study type |
| (Lee et al., 2014) | No usable data |
| (Lee et al., 2016) | No usable data |
| (Lee et al., 2018) | Data not accessible |
| (ley et al., 2018) | No usable data |
| (Li et al., 2012) | No usable data |
| (Lieberman et al., 1991) | No usable data |
| (Lieberman et al., 1994) | No usable data |
| (Lindenmayer and Apergi, 1996) | Inappropriate study type |
| (Liu et al., 1996) | No usable data |
| (Liu et al., 2012) | No usable data |
| (Llorca et al., 2002) | No usable data |
| (Lopes et al., 2018) | No usable data |
| (Lu et al., 2000) | No usable data |
| (Lu et al., 2018) | No usable data |
| (Luisa Imaz et al., 2018) | No usable data |
| (Lustenberger et al., 2011) | Inappropriate study type |
| (Ma et al., 2018) | No usable data |
| (Ma et al., 2018) | No usable data |
| (Matsuda et al., 1996) | No usable data |
| (Mauri et al., 2003) | No usable data |
| (Mauri et al., 2004) | No usable data |
| (Mayerova et al., 2018) | No usable data |
| (McKean et al., 2008) | No usable data |
| (Meehan et al., 2019) | No usable data |
| (Meiklejohn et al., 2019) | No usable data |
| (Melent'ev and Kurochkina, 1999) | No usable data |
| (Melkersson and Hulting, 2001) | No usable data |
| (Melkersson et al., 1999) | No usable data |
| (Melkersson et al., 2007) | No usable data |
| (Meltzer et al., 1989) | Inappropriate study type |
| (Meltzer et al., 1995) | Incorrect patient population |
| (Meltzer, 1992) | Inappropriate study type |
| (Meltzer, 2015) | No usable data |
| (Melzer-Ribeiro et al., 2015) | No usable data |
| (Menkes et al., 2018) | No usable data |
| (Mennickent et al., 2007) | No usable data |
| (Mennickent et al., 2010) | No usable data |
| (Merli and Haefele, 2011) | No usable data |
| (Meyer et al., 1996) | No usable data |
| (Meyer, 2001) | No usable data |
| (MHRA, 2021) | Inappropriate study type |
| (Miller et al., 1994) | Data not accessible |
| (Miller, 1991) | No usable data |
| (Miller, 1996) | Inappropriate study type |
| (Molins et al., 2017) | No usable data |
| (Morozova et al., 2018) | Data not accessible |
| (Mouaffak et al., 2006) | Inappropriate study type |
| (Mowerman and Siris, 1996) | No usable data |
| (Munro et al., 2004) | No usable data |
| (Nagendrappa et al., 2019) | No usable data |
| (Ng et al., 2005) | No usable data |
| (Nijenhuis and Van Rhenen, 2016) | No usable data |
| (Normann et al., 1998) | Inappropriate study type |
| (Olesen, 1998) | Inappropriate study type |
| (Oloyede et al., 2020) | No usable data |
| (Ordonez et al., 2014) | Data not accessible |
| (Palego et al., 2002) | No usable data |
| (Patteet et al., 2014) | No usable data |
| (Pauls et al., 2018) | No usable data |
| (Perry et al., 1998) | No usable data |
| (Pinzon Espinosa et al., 2017) | No usable data |
| (Piwowarska et al., 2016) | No usable data |
| (Potanin et al., 2015) | No usable data |
| (Préterre, 1995) | Data not accessible |
| (Prisco et al., 2015) | Data not accessible |
| (Qiu et al., 2015) | No usable data |
| (Raaska and Neuvonen, 2000) | No usable data |
| (Rajkumar et al., 2011) | No usable data |
| (Rajkumar et al., 2013) | No usable data |
| (Renne et al., 2016) | No usable data |
| (Repo-Tiihonen et al., 2005) | No usable data |
| (Rostami-Hodjegan et al., 2004) | No usable data |
| (Saito et al., 2016) | No usable data |
| (Salazar-Pereyra et al., 2011) | No usable data |
| (Samanaite et al., 2018) | Inappropriate study type |
| (Schirmbeck and Zink, 2013) | Inappropriate study type |
| (Schoretsanitis et al., 2019) | Inappropriate study type |
| (Schoretsanitis et al., 2020) | No usable data |
| (Schoretsanitis et al., 2021) | No usable data |
| (Schulte, 2003) | Inappropriate study type |
| (Schulz et al., 1995) | No usable data |
| (Seppala et al., 1999) | No usable data |
| (Shang et al., 2014) | No usable data |
| (Singh et al., 2015) | No usable data |
| (Spina et al., 1998) | No usable data |
| (Sporn et al., 2007) | No usable data |
| (Stahel et al., 2016) | No usable data |
| (Stark and Scott, 2012) | Inappropriate study type |
| (Stieffenhofer et al., 2011a) | No usable data |
| (Stieffenhofer et al., 2011b) | Data not accessible |
| (Subramaniam et al., 2007) | No usable data |
| (Suhas et al., 2020) | No usable data |
| (Suzuki et al., 2011) | Inappropriate study type |
| (Tang et al., 2007) | No usable data |
| (Taylor et al., 2011) | No usable data |
| (Thornton et al., 2015) | Inappropriate study type |
| (Thorup and Fog, 1977) | No usable data |
| (Tsuda et al., 2014) | Inappropriate study type |
| (Turjap and Jurica, 2013) | No usable data |
| (Ulrich et al., 2003) | No usable data |
| (Vailleau et al., 1996) | No usable data |
| (Vallette et al., 2001) | Incorrect patient population |
| (van der Weide et al., 2003) | No usable data |
| (VanderZwaag et al., 1996) | No usable data |
| (Verma et al., 2021) | No usable data |
| (Vincent and Halme, 2011) | No usable data |
| (Wang and Li, 2012) | Inappropriate study type |
| (Wehring et al., 2018) | No usable data |
| (Wenzel-Seifert et al., 2011) | No usable data |
| (Wilborn et al., 2011) | Data not accessible |
| (Wohkittel et al., 2016) | No usable data |
| (Woldeyohannes et al., 2017) | No usable data |
| (Wong et al., 2006) | No usable data |
| (Xiang et al., 2006) | No usable data |
| (Yada et al., 2021) | No usable data |
| (Yağcioğlu et al., 2005) | No usable data |
| (Yamaguchi et al., 2009) | No usable data |
| (Yamaguchi et al., 2009) | No usable data |
| (Yelmo-Cruz et al., 2016) | No usable data |
| (Youn et al., 2016) | No usable data |
| (Yuan et al., 2002) | No usable data |
| (Yuanguang et al., 1998) | No usable data |
| (Yue et al., 2005) | Data not accessible |
| (Zai et al., 2012) | No usable data |
| (Zhou et al., 2009) | No usable data |
| (Zivkovic et al., 2009) | No usable data |

Bibliography:

ACKENHEIL, M., BRAU, H., BURKHART, A., FRANKE, A. & PACHA, W. 1976. [Antipsychotic efficacy in relation to plasma levels of clozapine (author's transl)]. *Arzneimittelforschung,* 26**,** 1156-8.

ADEJUMO, S. A., ONIOSUN, B., AKPOILIH, O. A., ADESEKO, A. & AROWO, D. O. 2021. Anatomical changes, osmolytes accumulation and distribution in the native plants growing on Pb-contaminated sites. *Environ Geochem Health,* 43**,** 1537-1549.

ADLER, G., GRIESHABER, S., FAUDE, V., THEBALDI, B. & DRESSING, H. 2002. Clozapine in patients with chronic schizophrenia: serum level, EEG and memory performance. *Pharmacopsychiatry,* 35**,** 190-4.

AISSA, A., JOUINI, R., OUALI, U., ZGUEB, Y., NACEF, F. & EL HECHMI, Z. 2022. Clinical predictors of response to clozapine in Tunisian patients with treatment resistant schizophrenia. *Compr Psychiatry,* 112**,** 152280.

AKAMINE, Y., SUGAWARA-KIKUCHI, Y., UNO, T., SHIMIZU, T. & MIURA, M. 2017. Quantification of the steady-state plasma concentrations of clozapine and N-desmethylclozapine in Japanese patients with schizophrenia using a novel HPLC method and the effects of CYPs and ABC transporters polymorphisms. *Ann Clin Biochem,* 54**,** 677-685.

ALBERS, L. J. & OZDEMIR, V. 2004. Pharmacogenomic-guided rational therapeutic drug monitoring: Conceptual framework and application platforms for typical antipsychotics. *Current Medicinal Chemistry,* 11**,** 297-312.

ALDERMAN, C. P. 2004. Developments in psychiatry - 2004. *Journal of Pharmacy Practice and Research,* 34**,** 149-151.

ALLY, B. & ALLY, M. 2015. Clozapine in the community. *Australian Journal of Pharmacy,* 96**,** 66-71.

AMBASTA, A., CARSON, J. & CHURCH, D. L. 2015. The use of biomarkers and molecular methods for the earlier diagnosis of invasive aspergillosis in immunocompromised patients. *Med Mycol,* 53**,** 531-57.

ARANGO, C. 2019. The optimise study: Amisulpride and olanzapine followed by openlabel treatment with clozapine in first-episode schizophrenia. *Schizophrenia Bulletin,* 45**,** S115.

ASENJO LOBOS, C., KOMOSSA, K., RUMMEL-KLUGE, C., HUNGER, H., SCHMID, F., SCHWARZ, S. & LEUCHT, S. 2010. Clozapine versus other atypical antipsychotics for schizophrenia. *Cochrane Database Syst Rev***,** CD006633.

ASKARI, G., NASIRI, M., MOZAFFARI-KHOSRAVI, H., REZAIE, M., BAGHERI-BIDAKHAVIDI, M. & SADEGHI, O. 2017. The effects of folic acid and pyridoxine supplementation on characteristics of migraine attacks in migraine patients with aura: A double-blind, randomized placebo-controlled, clinical trial. *Nutrition,* 38**,** 74-79.

ASSION, H. J., REINBOLD, H., LEMANSKI, S., BASILOWSKI, M. & JUCKEL, G. 2008. Amisulpride augmentation in patients with schizophrenia partially responsive or unresponsive to clozapine. A randomized, double-blind, placebo-controlled trial. *Pharmacopsychiatry,* 41**,** 24-8.

ATMACA, M., KULOGLU, M., TEZCAN, E. & USTUNDAG, B. 2003. Serum leptin and triglyceride levels in patients on treatment with atypical antipsychotics. *J Clin Psychiatry,* 64**,** 598-604.

AVILES-GONZALEZ, L., DUTTON, T. & GRIFFITH, J. 2013. Assessment of the utility of clozapine levels in veterans at the tuscaloosa VA medical center. *Journal of Pharmacy Practice,* 26**,** 320.

AXELSSON, R. 1998. Clinical utility of antipsychotic drug monitoring in schizophrenic patients. *Nordic Journal of Psychiatry, Supplement,* 52**,** 68.

AYMARD, N., BALDACCI, C., LEYRIS, A., SMAGGHE, P. O., TRIBOLET, S., VACHERON, M. N., VIALA, A. & CAROLI, F. 1997. Neuroleptic-resistant schizophrenic patients treated by clozapine: clinical evolution, plasma and red blood cell clozapine and desmethylclozapine levels. *Therapie,* 52**,** 227-32.

AYMARD, N., VIALA, A., BALDACCI, C., SMAGGHE, P. O., VACHERON, M. N. & CAROLI, F. 1999. Pharmacoclinical strategy in neuroleptic resistant schizophrenic patients treated by clozapine: clinical evolution, concentration of plasma and red blood cell clozapine and desmethylclozapine, whole blood serotonin and tryptophan. *Prog Neuropsychopharmacol Biol Psychiatry,* 23**,** 25-41.

BABLENIS, E., WEBER, S. S. & WAGNER, R. L. 1989. Clozapine: a novel antipsychotic agent. *DICP,* 23**,** 109-15.

BAKER, M. & WHITE, T. 2004. Life after clozapine. *Med Sci Law,* 44**,** 217-21.

BALANT-GORGIA, A. E., BALANT, L. P. & ANDREOLI, A. 1993. Pharmacokinetic optimisation of the treatment of psychosis. *Clin Pharmacokinet,* 25**,** 217-36.

BALDELLI, S., CHELI, S., MONTRASIO, C., CATTANEO, D. & CLEMENTI, E. 2020. Therapeutic drug monitoring and pharmacogenetics of antipsychotics and antidepressants in real life settings: A 5-year single centre experience. *World J Biol Psychiatry***,** 1-12.

BALDESSARINI, R. J. & FRANKENBURG, F. R. 1994. 'Clozapine: A novel antipsychotic agent': Reply. *The New England Journal of Medicine,* 331**,** 276-276.

BARNES, T. R. 2011. Evidence-based guidelines for the pharmacological treatment of schizophrenia: Recommendations from the British Association for Psychopharmacology. *Journal of Psychopharmacology,* 25**,** 567-620.

BARNES, T. R., LEESON, V. C., PATON, C., MARSTON, L., DAVIES, L., WHITTAKER, W., OSBORN, D., KUMAR, R., KEOWN, P., ZAFAR, R., IQBAL, K., SINGH, V., FRIDRICH, P., FITZGERALD, Z., BAGALKOTE, H., HADDAD, P. M., HUSNI, M. & AMOS, T. 2017. Amisulpride augmentation in clozapine-unresponsive schizophrenia (AMICUS): a double-blind, placebo-controlled, randomised trial of clinical effectiveness and cost-effectiveness. *Health Technol Assess,* 21**,** 1-56.

BARTELS, M. 1992. Pharmacotherapy of schizophrenia in Germany. *Keio J Med,* 41**,** 59-63.

BAUMANN, P. 2008. Pharmacovigilance in psychiatry: pharmacogenetic tests and therapeutic drug monitoring are promising tools. *Expert Rev Clin Pharmacol,* 1**,** 183-5.

BECHELLI, L. P., BASTOS, O. C., BUSNELLO, E. A. & CAETANO, D. 1994. Clozapina no Brasil—Avaliaćão da aplicabilidade do sistema de farmacovigilância e resultados terapêuticos preliminares = Clozapine in Brazil: Evaluation of the feasibility of the drug surveillance system and preliminary therapeutic results. *Jornal Brasileiro de Psiquiatria,* 43**,** 389-399.

BELL, R., MCLAREN, A., GALANOS, J. & COPOLOV, D. 1998. The clinical use of plasma clozapine levels. *Aust N Z J Psychiatry,* 32**,** 567-74.

BENNETT, D. M., BREMNER, M. & GRAY, C. 2008. The clinical use of plasma clozapine levels in a maximum security setting. *Int J Psychiatry Clin Pract,* 12**,** 228-34.

BENNETT, J. A. & KECK, P. E., JR. 1996. A target-dose finding study of clozapine in patients with schizophrenia. *Ann Clin Psychiatry,* 8**,** 19-21.

BENVENUTI, A., LATTANZI, L., BARTOLOMMEI, N., LUCHINI, F., COSENTINO, L. & MAURI, M. 2012. Ziprasidone as adjunctive therapy in patients with schizoaffective or bipolar disorder treated with clozapine. *European Psychiatry. Conference: 20th European Congress of Psychiatry, EPA,* 27.

BHATTACHARYA, R., WHITE, L. & PISANESCHI, L. 2021. Clozapine prescribing: comparison of clozapine dosage and plasma levels between White British and Bangladeshi patients. *BJPsych Bull,* 45**,** 22-27.

BONDOLFI, G., DUFOUR, H., PATRIS, M., MAY, J. P., BILLETER, U., EAP, C. B. & BAUMANN, P. 1998. Risperidone versus clozapine in treatment-resistant chronic schizophrenia: a randomized double-blind study. The Risperidone Study Group. *Am J Psychiatry,* 155**,** 499-504.

BOOKHOLT, D. E. & BOGERS, J. P. 2014. Oral contraceptives raise plasma clozapine concentrations. *J Clin Psychopharmacol,* 34**,** 389-90.

BOWSKILL, S., COUCHMAN, L., MACCABE, J. H. & FLANAGAN, R. J. 2012. Plasma clozapine and norclozapine in relation to prescribed dose and other factors in patients aged 65 years and over: data from a therapeutic drug monitoring service, 1996-2010. *Hum Psychopharmacol,* 27**,** 277-83.

BRAGA, R. J., MENDELOWITZ, A. J., FINK, M., SCHOOLER, N. R., BAILINE, S. H., MALUR, C. & PETRIDES, G. 2009. A randomized controlled trial of ECT in clozapine-refractory schizophrenia. *Biological Psychiatry,* 1**,** 212S-213S.

BRAU, H., BURKHART, A., PACHA, W. & ACKENHEIL, M. 1978. [Relationships between effects and plasma levels of clozapine (author's transl)]. *Arzneimittelforschung,* 28**,** 1300.

BRUSOV, O. S., KOLIASKINA, G. I., KALEDA, V. G., SEKIRINA, T. P., KUSHNER, S. G. & VASIL'EVA, E. F. 2010. [The use of canonical correlation analysis for the evaluation of correlation strength between clinical and biological parameters]. *Zh Nevrol Psikhiatr Im S S Korsakova,* 110**,** 11-9.

BUCKLEY, P. F., COLA, P., HASEGAWA, M., LYS, C. & THOMPSON, P. 1997. Clozapine plasma levels and dosing strategies in patients with treatment-refractory schizophrenia. *Irish Journal of Psychological Medicine,* 14**,** 85-88.

BUSTILLO, M., ZABALA, A., QUEREJETA, I., CARTON, J. I., MENTXAKA, O., GONZALEZ-PINTO, A., GARCIA, S., MEANA, J. J., EGUILUZ, J. I. & SEGARRA, R. 2018. Therapeutic Drug Monitoring of Second-Generation Antipsychotics for the Estimation of Early Drug Effect in First-Episode Psychosis: A Cross-sectional Assessment. *Ther Drug Monit,* 40**,** 257-267.

CARCELLER-SINDREU, M., PORTELLA, M. J., CARMONA, C., RAMETTI, G., PUIGDEMONT, D., FIGUERAS, M., FERNANDEZ-VIDAL, A., VILLALTA, L. & ALVAREZ, E. 2014. Neuropsychological effects of maintenance treatment with Clozapine in Treatment-Resistant Psychotic Disorder. *Actas Esp Psiquiatr,* 42**,** 68-73.

CARULLI, G., BUDA, G., AZZARA, A., CIANCIA, E. M., SAMMURI, P., DOMENICHINI, C., GUERRI, V. & PETRINI, M. 2016. CD229 Expression on Bone Marrow Plasma Cells from Patients with Multiple Myeloma and Monoclonal Gammopathies of Uncertain Significance. *Acta Haematol,* 135**,** 11-4.

CASTBERG, I., WESTIN, A. A., SKOGVOLL, E. & SPIGSET, O. 2017. Effects of age and gender on the serum levels of clozapine, olanzapine, risperidone, and quetiapine. *Acta Psychiatr Scand,* 136**,** 455-464.

CATALAN, R., PENADES, R., VAZQUEZ, M., TORRA, M., MASANA, G., PONS, A. & BERNARDO, M. 2012. The pharmacokinetic profile of CLZ might explain the variability in the acute response in treatment refractory schizophrenia. *Schizophrenia Research,* 1**,** S259-S260.

CATALAN, R., VAZQUEZ, M., PONS, A., MASANA, G., PENADES, R. & BERNARDO, M. 2011. Implications of clozaping and desmethylclozapine blood levels to clinical response in treatment refractory schizophrenia. *Schizophrenia Bulletin,* 37**,** 286-287.

CENTORRINO, F., BALDESSARINI, R. J., KANDO, J., FRANKENBURG, F. R., VOLPICELLI, S. A., PUOPOLO, P. R. & FLOOD, J. G. 1994. Serum concentrations of clozapine and its major metabolites: effects of cotreatment with fluoxetine or valproate. *Am J Psychiatry,* 151**,** 123-5.

CESKOVA, E. 1995. Plasma levels of clozapine. [Czech]. *Ceska a slovenska psychiatrie / Ceska lekarska spolecnost J.E***,** 234-240.

CHANG, J. S., AHN, Y. M., PARK, H. J., LEE, K. Y., KIM, S. H., KANG, U. G. & KIM, Y. S. 2008. Aripiprazole augmentation in clozapine-treated patients with refractory schizophrenia: an 8-week, randomized, double-blind, placebo-controlled trial. *J Clin Psychiatry,* 69**,** 720-31.

CHANG, J. S., LEE, N. Y., AHN, Y. M. & KIM, Y. S. 2012. The sustained effects of aripiprazole-augmented clozapine treatment on the psychotic symptoms and metabolic profiles of patients with refractory schizophrenia. *J Clin Psychopharmacol,* 32**,** 282-4.

CHANG, W. H., LIN, S. K., LANE, H. Y., HU, W. H., JANN, M. W. & LIN, H. N. 1997. Clozapine dosages and plasma drug concentrations. *J Formos Med Assoc,* 96**,** 599-605.

CHARFI, O. 2013. Therapeutic drug monitoring of clozapine in a Tunisian population of patient with psychiatric disorders. *Fundamental and Clinical Pharmacology,* 1**,** 67-68.

CHONG, S. A. & REMINGTON, G. 1998. Re: Sertraline-clozapine interaction. *Can J Psychiatry,* 43**,** 856-7.

CHUNG, C. & REMINGTON, G. 2005. Predictors and markers of clozapine response. *Psychopharmacology (Berl),* 179**,** 317-35.

CHUNG, I. W., KIM, H. S., YOUNG, T., JEONG, S. H., JUNG, H. Y. & KIM, Y. S. 2019a. P.068 Association between electroencephalogram changes and plasma clozapine levels in clozapine-treated patients. *European Neuropsychopharmacology,* 29**,** S66-S67.

CHUNG, I. W., YOUN, T., KIM, H. S. & KIM, Y. S. 2019b. Association of EEG abnormalities and plasma clozapine levels in patients with major psychotic disorders. *European Neuropsychopharmacology,* 29**,** S242-S243.

CITROME, L. & VOLAVKA, J. 2002. Optimal dosing of atypical antipsychotics in adults: a review of the current evidence. *Harv Rev Psychiatry,* 10**,** 280-91.

COSTA-DOOKHAN, K. A., AGARWAL, S. M., CHINTOH, A., TRAN, V. N., STOGIOS, N., EBDRUP, B. H., SOCKALINGAM, S., RAJJI, T. K., REMINGTON, G. J., SISKIND, D. & HAHN, M. K. 2020. The clozapine to norclozapine ratio: a narrative review of the clinical utility to minimize metabolic risk and enhance clozapine efficacy. *Expert Opin Drug Saf,* 19**,** 43-57.

COSTA-DOOKHAN, K. A., RAJJI, T. K., TRAN, V. N., BOWDEN, S., MUELLER, D. J., REMINGTON, G. J., AGARWAL, S. M. & HAHN, M. K. 2021. Associations between plasma clozapine/N-desmethylclozapine ratio, insulin resistance and cognitive performance in patients with co-morbid obesity and ultra-treatment resistant schizophrenia. *Sci Rep,* 11**,** 2004.

COUCHMAN, L., BOWSKILL, S. V., MACCABE, J. H., PATEL, M. X. & FLANAGAN, R. J. 2012. Sex differences in plasma clozapine/norclozapine and olanzapine concentrations in clinical practice: Data from a therapeutic drugmonitoring service. *Schizophrenia Research,* 1**,** S186.

COUCHMAN, L., MILLER, J., THANABALASINGAM, K., FLANAGAN, R. J. & HEALD, A. H. 2015. Sex differences in clozapine metabolism may explain in part the higher incidence of diabetes and weight gain in women given this drug. *Diabetic Medicine,* 1**,** 58.

COUCHMAN, L., MORGAN, P. E., SPENCER, E. P. & FLANAGAN, R. J. 2010. Plasma clozapine, norclozapine, and the clozapine:norclozapine ratio in relation to prescribed dose and other factors: data from a therapeutic drug monitoring service, 1993-2007. *Ther Drug Monit,* 32**,** 438-47.

DAL SANTO, F., JARRATT-BARNHAM, I., GONZALEZ-BLANCO, L., GARCIA-PORTILLA, M. P., BOBES, J. & FERNANDEZ-EGEA, E. 2020. Longitudinal effects of clozapine concentration and clozapine to N-desmethylclozapine ratio on cognition: A mediation model. *Eur Neuropsychopharmacol,* 33**,** 158-163.

DE LEON, J., RAJKUMAR, A. P., KAITHI, A. R., SCHORETSANITIS, G., KANE, J. M., WANG, C. Y., TANG, Y. L., LIN, S. K., HONG, K. S., FAROOQ, S., NG, C. H., RUAN, C. J. & ANDRADE, C. 2020a. Do Asian Patients Require Only Half of the Clozapine Dose Prescribed for Caucasians? A Critical Overview. *Indian J Psychol Med,* 42**,** 4-10.

DE LEON, J., RUAN, C. J., SCHORETSANITIS, G. & DE LAS CUEVAS, C. 2020b. A Rational Use of Clozapine Based on Adverse Drug Reactions, Pharmacokinetics, and Clinical Pharmacopsychology. *Psychother Psychosom,* 89**,** 200-214.

DE LEON, J., SCHORETSANITIS, G., KANE, J. M. & RUAN, C. J. 2020c. Using therapeutic drug monitoring to personalize clozapine dosing in Asians. *Asia Pac Psychiatry,* 12**,** e12384.

DE LORENZO, L., MERCHAN, F., LAPORTE, P., THOMPSON, R., CLARKE, J., SOUSA, C. & CRESPI, M. 2009. A novel plant leucine-rich repeat receptor kinase regulates the response of Medicago truncatula roots to salt stress. *Plant Cell,* 21**,** 668-80.

DE LUCA, V., DANIELA, S. S., BORLIDO, C., POWELL, V., LEAH, B., ROSHNI, P. & REMINGTON, G. 2019a. Admixture analysis to model clozapine pharmacokinetics: Comparison of two hospital-based samples. *Schizophrenia Bulletin,* 45**,** S348-S349.

DE LUCA, V., TAKEUCHI, H., BORLIDO, C., POWELL, V., BURTON, L., SONG, J., LUNDY, J. & REMINGTON, G. 2019b. Admixture analysis for clozapine plasma level monitoring to optimize treatment response. *European Neuropsychopharmacology,* 29**,** S286-S287.

DE OLIVEIRA MARQUES, F., OLIVEIRA, S. A., DE LIMA, E. S. P. F., NOJOZA, W. G., DA SILVA SENA, M., FERREIRA, T. M., COSTA, B. G. & LIBORIO, A. B. 2017. Kinetic estimated glomerular filtration rate in critically ill patients: beyond the acute kidney injury severity classification system. *Crit Care,* 21**,** 280.

DERVAUX, A. & LAQUEILLE, X. 2007. Treatment of tobacco use in smokers with schizophrenia. [French]. *Encephale,* 33**,** 629-632.

DETTLING, M., SACHSE, C., BROCKMÖLLER, J., SCHLEY, J., MÜLLER-OERLINGHAUSEN, B., PICKERSGILL, I., ROLFS, A., SCHAUB, R. T. & SCHMIDER, J. 2000. Long-term therapeutic drug monitoring of clozapine and metabolites in psychiatric in- and outpatients. *Psychopharmacology (Berl),* 152**,** 80-6.

DIAZ, F. J., JOSIASSEN, R. C. & DE LEON, J. 2018. The Effect of Body Weight Changes on Total Plasma Clozapine Concentrations Determined by Applying a Statistical Model to the Data From a Double-Blind Trial. *J Clin Psychopharmacol,* 38**,** 442-446.

DOBRINAS, M., CORNUZ, J., ONEDA, B., KOHLER SERRA, M., PUHL, M. & EAP, C. B. 2011. Cytochrome P450 1A2 activity and inducibility: Impact of smoking, smoking cessation and genetic polymorphisms. *Therapeutic Drug Monitoring,* 33**,** 473.

DUMORTIER, G., LOCHU, A., ZERROUK, A., VAN NIEUWENHUYSE, V., COLEN DE MELO, P., ROCHE RABREAU, D. & DEGRASSAT, K. 1998. Whole saliva and plasma levels of clozapine and desmethylclozapine. *J Clin Pharm Ther,* 23**,** 35-40.

DZIEDZICKA-WASYLEWSKA, M., FARON-GORECKA, A., GORECKI, A. & KUSMIDER, M. 2008. Mechanism of action of clozapine in the context of dopamine D<inf>1</inf>-D<inf>2</inf> receptor hetero-dimerization - A working hypothesis. *Pharmacological Reports,* 60**,** 581-587.

ENGLISCH, S. & ZINK, M. 2008. Combined antipsychotic treatment involving clozapine and aripiprazole. *Prog Neuropsychopharmacol Biol Psychiatry,* 32**,** 1386-92.

ESSLINGER, C., INTA, D., ENGLISCH, S., ESSER, A. & ZINK, M. 2010. Clozapine combined with paliperidone observations in schizophrenic patients with insufficient responses to clozapine monotherapy. *German Journal of Psychiatry,* 13**,** 37-40.

FABRAZZO, M., ESPOSITO, G., FUSCO, R. & MAJ, M. 1996. Effect of treatment duration on plasma levels of clozapine and N-desmethylclozapine in men and women. *Psychopharmacology (Berl),* 124**,** 197-200.

FABRAZZO, M., LA PIA, S., MONTELEONE, P., ESPOSITO, G., PINTO, A., DE SIMONE, L., BENCIVENGA, R. & MAJ, M. 2002. Is the Time Course of Clozapine Response Correlated to the Time Course of Clozapine Plasma Levels? A One-Year Prospective Study in Drug-Resistant Patients with Schizophrenia. *Neuropsychopharmacology,* 27**,** 1050-1055.

FLEISCHHAKER, C., SCHULZ, E. & REMSCHMIDT, H. 1998. [Depressive symptoms and biogenic amines as predictors of treatment response in schizophrenic illnesses in childhood and adolescence]. *Z Kinder Jugendpsychiatr Psychother,* 26**,** 235-43.

FRAZIER, J. A., COHEN, L. G., JACOBSEN, L., GROTHE, D., FLOOD, J., BALDESSARINI, R. J., PISCITELLI, S., KIM, G. S. & RAPOPORT, J. L. 2003. Clozapine pharmacokinetics in children and adolescents with childhood-onset schizophrenia. *J Clin Psychopharmacol,* 23**,** 87-91.

GAERTNER, I., GAERTNER, H. J., VONTHEIN, R. & DIETZ, K. 2001. Therapeutic drug monitoring of clozapine in relapse prevention: a five-year prospective study. *J Clin Psychopharmacol,* 21**,** 305-10.

GOLDEN, G. & HONIGFELD, G. 2008. Bioequivalence of clozapine orally disintegrating 100-mg tablets compared with clozapine solid oral 100-mg tablets after multiple doses in patients with schizophrenia. *Clin Drug Investig,* 28**,** 231-9.

GOLDSMITH, D., KOPELOVICH, S., NOVACEK, D., WIDENER, J., WOMMACK, E., FELGER, J., MILLER, A. & COTES, R. 2017. Association between inflammatory markers and negative symptoms in individuals with persistent symptoms of schizophrenia treated with clozapine. *Neuropsychopharmacology,* 41**,** S390-S391.

GONZALEZ-ESQUIVEL, D. F., CASTRO, N., RAMIREZ-BERMUDEZ, J., CUSTODIO, V., ROJAS-TOME, S., CASTRO-ROMAN, R. & JUNG-COOK, H. 2011. Plasma levels of clozapine and norclozapine in Mexican schizophrenia patients. *Arzneimittelforschung,* 61**,** 335-9.

GRABSKI, B. & KASPAREK, K. 2017. Sexual problems in homo- and bisexual men - the context of the issue. *Psychiatr Pol,* 51**,** 75-83.

GREENWOOD-SMITH, C., LUBMAN, D. I. & CASTLE, D. J. 2003. Serum clozapine levels: a review of their clinical utility. *J Psychopharmacol,* 17**,** 234-8.

GRILLI-TISSOT, M. C. R. & LOUZA, M. R. 2014. Treatment of clozapine-induced hypersalivation with amisulpride: A systematic review. *Schizophrenia Research,* 1**,** S190.

GRILLO, R. W., OTTONI, G. L., LEKE, R., SOUZA, D. O., PORTELA, L. V. & LARA, D. R. 2007. Reduced serum BDNF levels in schizophrenic patients on clozapine or typical antipsychotics. *J Psychiatr Res,* 41**,** 31-5.

GRUNDER, G., LANDVOGT, C., VERNALEKEN, I., BUCHHOLZ, H. G., ONDRACEK, J., SIESSMEIER, T., HARTTER, S., SCHRECKENBERGER, M., STOETER, P., HIEMKE, C., ROSCH, F., WONG, D. F. & BARTENSTEIN, P. 2006. The striatal and extrastriatal D2/D3 receptor-binding profile of clozapine in patients with schizophrenia. *Neuropsychopharmacology,* 31**,** 1027-35.

GRUNDMANN, M., KACIROVA, I. & URINOVSKA, R. 2014. Therapeutic drug monitoring of atypical antipsychotic drugs. *Acta Pharm,* 64**,** 387-401.

GUITTON, C., ABBAR, M., KINOWSKI, J. M., CHABRAND, P. & BRESSOLLE, F. 1998a. Multiple-dose pharmacokinetics of clozapine in patients with chronic schizophrenia. *J Clin Psychopharmacol,* 18**,** 470-6.

GUITTON, C., KINOWSKI, J. M., GOMENI, R. & BRESSOLLE, F. 1998b. A Kinetic Model for Simultaneous Fit of Clozapine and Norclozapine Concentrations in Chronic Schizophrenic Patients during Long-Term Treatment. *Clin Drug Investig,* 16**,** 35-43.

GUNDUZ-BRUCE, H., OLIVER, S., GUEORGUIEVA, R., FORSELIUS-BIELEN, K., D'SOUZA, D. C., ZIMOLO, Z., TEK, C., KALIORA, S., RAY, S. & PETRIDES, G. 2013. Efficacy of pimozide augmentation for clozapine partial responders with schizophrenia. *Schizophr Res,* 143**,** 344-7.

HANTULIK, P., WITTIG, K., HENSCHEL, Y., OCHSE, J., VAHTERISTO, M. & RYTILA, P. 2015. Usage and usability of one dry powder inhaler compared to other inhalers at therapy start: an open, non-interventional observational study in Poland and Germany. *Pneumonol Alergol Pol,* 83**,** 365-77.

HARING, C., BARNAS, C., SARIA, A., HUMPEL, C. & FLEISCHHACKER, W. W. 1989a. Dose-related plasma levels of clozapine. *J Clin Psychopharmacol,* 9**,** 71-2.

HARING, C., FLEISCHHACKER, W. W., SCHETT, P., HUMPEL, C., BARNAS, C. & SARIA, A. 1990. Influence of patient-related variables on clozapine plasma levels. *Am J Psychiatry,* 147**,** 1471-5.

HARING, C., MEISE, U., HUMPEL, C., SARIA, A., FLEISCHHACKER, W. W. & HINTERHUBER, H. 1989b. Dose-related plasma levels of clozapine: influence of smoking behaviour, sex and age. *Psychopharmacology (Berl),* 99 Suppl**,** S38-40.

HARJANINGSIH, W., AYUNIRRAHMAH, L. & PUTRI, N. J. V. 2021. The therapeutic outcomes and adverse drug reactions study of Clozapine on Schizophrenia inpatients in the grhasia psychiatric hospital Yogyakarta, Indonesia. *Pharmacy Education,* 21**,** 287-295.

HART, X. M., HIRJAK, D., SCHARF, D., SCHWIND, N. & GRÜNDER, G. 2021. P.326 Clozapine pharmacokinetics and blood level fluctuations in smoking and nonsmoking patients with schizophrenia. *European Neuropsychopharmacology,* 44**,** S58.

HAVAKI-KONTAXAKI, B. J., FERENTINOS, P. P., KONTAXAKIS, V. P., PAPLOS, K. G. & SOLDATOS, C. R. 2006. Concurrent administration of clozapine and electroconvulsive therapy in clozapine-resistant schizophrenia. *Clin Neuropharmacol,* 29**,** 52-6.

HERMIDA, J., PAZ, E. & TUTOR, J. C. 2008. Clozapine and norclozapine concentrations in serum and plasma samples from schizophrenic patients. *Ther Drug Monit,* 30**,** 41-5.

HOIMARK, L., UHRSKOV SORENSEN, L. & VUKELIC ANDERSEN, L. 2020. Plasma concentrations of antipsychotics and QTc prolongation: a pilot study. *Nord J Psychiatry,* 74**,** 374-379.

HOTHAM, J. E., SIMPSON, P. J., BROOMAN-WHITE, R. S., BASU, A., ROSS, C. C., HUMPHREYS, S. A., LARKIN, F., GUPTA, N. & DAS, M. 2014. Augmentation of clozapine with amisulpride: an effective therapeutic strategy for violent treatment-resistant schizophrenia patients in a UK high-security hospital. *CNS Spectr,* 19**,** 403-10.

HUANG, H.-C., LUA, A. C., WU, L. S.-H., WU, B.-J., LEE, S.-M. & LIU, C.-Z. 2016. Cigarette smoking has a differential effect on the plasma level of clozapine in Taiwanese schizophrenic patients associated with the CYP1A2 gene—163A/C single nucleotide polymorphism. *Psychiatric Genetics,* 26**,** 172-177.

IGLESIAS GARCIA, C., IGLESIAS ALONSO, A. & BOBES, J. 2017. Concentrations in plasma clozapine levels in schizophrenic and schizoaffective patients. *Rev Psiquiatr Salud Ment,* 10**,** 192-196.

IGNJATOVIC RISTIC, D., COHEN, D., OBRADOVIC, A., NIKIC-DURICIC, K., DRASKOVIC, M. & HINIC, D. 2018. The Glasgow antipsychotic side-effects scale for clozapine in inpatients and outpatients with schizophrenia or schizoaffective disorder. *Nord J Psychiatry,* 72**,** 124-129.

JANN, M. W., LIU, H.-C., WEI, F.-C., LIN, S.-K., LIN, S.-K., HU, W.-H. & CHANG, W.-H. 1997. Gender differences in plasma clozapine levels and its metabolites in schizophrenic patients. *Human Psychopharmacology: Clinical and Experimental,* 12**,** 489-495.

JIANG, P., LIN, Z., JIN, Y., REN, J., LIU, H., CUI, H., WANG, J. & LI, C. 2016. Effect of Clonazepam Co-Administered with Clozapine on the Serum Clozapine and Norclozapine Concentration of Patients with Schizophrenia: A Retrospective Survey. *Shanghai Arch Psychiatry,* 28**,** 318-325.

JIANG, P., LIU, H. M., REN, J. J., LI, T., LIN, Z. G., JIN, Y., GUI, H. R., SUN, X. & LI, C. B. 2013. Detection of plasma clozapine concentration in psychosis disorder inpatients receiving comedications. [Chinese]. *Academic Journal of Second Military Medical University,* 34**,** 759-763.

JIN, H. 2014. China's private universities. *Science,* 346**,** 401.

JOHN, A. P. & KECANOVIC, A. 2021. Unusually high serum levels of clozapine associated with genetic polymorphism of CYP3A enzymes. *Asian J Psychiatr,* 57**,** 102126.

JONSDOTTIR, H., OPJORDSMOEN, S., BIRKENAES, A. B., ENGH, J. A., RINGEN, P. A., VASKINN, A., AAMO, T. O., FRIIS, S. & ANDREASSEN, O. A. 2010. Medication adherence in outpatients with severe mental disorders relation between self-reports and serum level. *Journal of Clinical Psychopharmacology,* 30**,** 169-175.

JONSSON, A. K., SPIGSET, O. & REIS, M. 2019. A Compilation of Serum Concentrations of 12 Antipsychotic Drugs in a Therapeutic Drug Monitoring Setting. *Ther Drug Monit,* 41**,** 348-356.

KALADJIAN, A., BERY, B., DETURMENY, E. & BRUGUEROLLE, B. 1999. Clozapine monitoring: plasma or serum levels? *Ther Drug Monit,* 21**,** 327-9.

KALTER, E., KONAS, S. & WEIZMAN, A. 2020. The Effect of Clozapine and Another Antipsychotic Combination Treatment on the Clinical Outcomes of Treatment Resistant Schizophrenia Inpatients. *Biological Psychiatry,* 87**,** S344.

KAUFMANN, A., RETTENBACHER, M. A., YALCIN, N., BIEDERMANN, F., EDLINGER, M., KEMMLER, G., WIDSCHWENDTER, C. G., TOENNES, S. W., FLEISCHHACKER, W. W. & HOFER, A. 2005. Expected and effective plasma concentrations of antipsychotics and changes in psychopathology. *European Neuropsychopharmacology,* 2**,** S486-S487.

KELLER, W. R., FISCHER, B. A., MCMAHON, R., MEYER, W., BLAKE, M. & BUCHANAN, R. W. 2014. Community adherence to schizophrenia treatment and safety monitoring guidelines. *J Nerv Ment Dis,* 202**,** 6-12.

KELLY, D., KEARNS, A. M., GLASSMAN, M., ATKINS, M. & MCQUIRE, P. 2020. Relevant clozapine blood levels using a point of care test (POCT). *Schizophrenia Bulletin,* 46**,** S309.

KELLY, D. L., VYAS, G., RICHARDSON, C. M., KOOLA, M., MCMAHON, R. P., BUCHANAN, R. W. & WEHRING, H. J. 2011. Adjunct minocycline to clozapine treated patients with persistent schizophrenia symptoms. *Schizophr Res,* 133**,** 257-8.

KESHAVARZI, F., FOX, T., WHISKEY, E. & TAYLOR, D. 2020. Change in plasma concentration of clozapine and norclozapine following a switch of oral formulation. *Ther Adv Psychopharmacol,* 10**,** 2045125319899263.

KHAN, A. Y. & PRESKORN, S. H. 2005. Examining concentration-dependent toxicity of clozapine: role of therapeutic drug monitoring. *J Psychiatr Pract,* 11**,** 289-301.

KIKUCHI, Y., ATAKA, K., YAGISAWA, K., OMORI, Y., KANBAYASHI, T. & SHIMIZU, T. 2015. Clozapine induced eeg abnormalities and the serum concentration of clozapine in Japanese patients with schizophrenia. *European Psychiatry,* 1**,** 901.

KIM, H. S., YOUN, T., KIM, S. H., JEONG, S. H., JUNG, H. Y., JEONG, S. W., KIM, K. K., KIM, Y. S. & CHUNG, I. W. 2019. Association between electroencephalogram changes and plasma clozapine levels in clozapine-treated patients. *Int Clin Psychopharmacol,* 34**,** 131-137.

KLAMPFL, K., MEHLER-WEX, C., WARNKE, A. & GERLACH, M. 2011. Therapeutic drug monitoring in child and adolescent psychiatry. [German]. *Psychopharmakotherapie,* 17**,** 193-200.

KOCAR, T., FREUDENMANN, R. W., SPITZER, M. & GRAF, H. 2018. Switching From Tobacco Smoking to Electronic Cigarettes and the Impact on Clozapine Levels. *J Clin Psychopharmacol,* 38**,** 528-529.

KOLA OYEWUMI, L., CERNOVSKY, Z. Z., FREEMAN, D. J. & STREINER, D. L. 2002. Relation of blood counts during clozapine treatment to serum concentrations of clozapine and nor-clozapine. *Canadian Journal of Psychiatry,* 47**,** 257-261.

KOOLE, M. I., ROELOFSEN, E. E., BAPTIST, E., VAN DER MEER, R., WILMS, E. B. & VISSER, L. E. 2019. The effect of a therapeutic guideline on the percentage of patients with supratherapeutic clozapine blood levels during hospital admission. [Dutch]. *Pharmaceutisch Weekblad,* 154**,** 27-32.

KRIVOY, A., WHISKEY, E., WEBB-WILSON, H., JOYCE, D., TRACY, D. K., GAUGHRAN, F., MACCABE, J. H. & SHERGILL, S. S. 2021. Outcomes in treatment-resistant schizophrenia: symptoms, function and clozapine plasma concentrations. *Ther Adv Psychopharmacol,* 11**,** 20451253211037179.

KURKAR, M., POYNTON, A., KLIMACH, J. & VERMA, J. 2015. When do psychiatrists request clozapine serum levels assays? Audit of the practice of psychiatrists in 3 hospitals in Manchester, UK. *European Psychiatry,* 1**,** 1601.

KURZ, M., HUMMER, M., KEMMLER, G., KURZTHALER, I., SARIA, A. & FLEISCHHACKER, W. W. 1998. Long-term pharmacokinetics of clozapine. *Br J Psychiatry,* 173**,** 341-4.

LAMMERS, C. H., DEUSCHLE, M., WEIGMANN, H., HARTTER, S., HIEMKE, C., HEESE, C. & HEUSER, I. 1999. Coadministration of clozapine and fluvoxamine in psychotic patients--clinical experience. *Pharmacopsychiatry,* 32**,** 76-7.

LANE, H. Y., CHANG, Y. C., CHANG, W. H., LIN, S. K., TSENG, Y. T. & JANN, M. W. 1999. Effects of gender and age on plasma levels of clozapine and its metabolites: analyzed by critical statistics. *J Clin Psychiatry,* 60**,** 36-40.

LANE, H. Y., JANN, M. W., CHANG, Y. C., CHIU, C. C., HUANG, M. C., LEE, S. H. & CHANG, W. H. 2001. Repeated ingestion of grapefruit juice does not alter clozapine's steady-state plasma levels, effectiveness, and tolerability. *J Clin Psychiatry,* 62**,** 812-7.

LANGREE, B., BATAIL, J. M., LEMAITRE, F., BLEHER, S., BURGOT, G., DRAPIER, D., VERDIER, M. C. & BELISSANT, E. 2013. Why 24 refractory schizophrenic patients well tolerate olanzapine up to 100 mg daily? The part of pharmacokinetic explanations. *Fundamental and Clinical Pharmacology,* 1**,** 68.

LATTUADA, E., COCCHI, F., ROSSATTINI, M., SOLDARINI, A., GENTILINI, G., DORIGATTI, F. & CAVALLARO, R. 2004. Brand-name versus generic clozapine for the treatment of resistant schizophrenia: Bioequivalence and interchangeability issues. [Italian]. *Italian Journal of Psychopathology,* 10**,** 267-271.

LEE, J., BHALOO, A., POWELL, V. & REMINGTON, G. 2014. Therapeutic drug monitoring of clozapine: Sampling error as a source of variance. *Therapeutic Drug Monitoring,* 36**,** 829-831.

LEE, J., BIES, R., TAKEUCHI, H., FERVAHA, G., BHALOO, A., POWELL, V. & REMINGTON, G. 2016. Quantifying intraindividual variations in plasma clozapine levels: a population pharmacokinetic approach. *J Clin Psychiatry,* 77**,** 681-7.

LEE, J., YEE, J. Y., SEE, Y. M., TANG, C., NG, B. T., REMINGTON, G. & CHOWBAY, B. 2018. Utility of saliva for monitoring of clozapine levels. *Schizophrenia Bulletin,* 44**,** S309.

LEE, S. T., RYU, S., KIM, S. R., KIM, M. J., KIM, S., KIM, J. W., LEE, S. Y. & HONG, K. S. 2012. Association study of 27 annotated genes for clozapine pharmacogenetics: validation of preexisting studies and identification of a new candidate gene, ABCB1, for treatment response. *J Clin Psychopharmacol,* 32**,** 441-8.

LEE, S. T., RYU, S., NAM, H. J., LEE, S. Y. & HONG, K. S. 2009. Determination of pharmacokinetic properties of clozapine and norclozapine in Korean schizophrenia patients. *Int Clin Psychopharmacol,* 24**,** 139-44.

LEY, S. A., SILK, S. W., FISHER, D. S., SUBRAMANIAM, K. & FLANAGAN, R. J. 2018. Clozapine and norclozapine concentrations in paired human plasma and serum samples. *Therapeutic Drug Monitoring,* 40**,** 148-150.

LI, L. J., SHANG, D. W., LI, W. B., GUO, W., WANG, X. P., REN, Y. P., LI, A. N., FU, P. X., JI, S. M., LU, W. & WANG, C. Y. 2012. Population pharmacokinetics of clozapine and its primary metabolite norclozapine in Chinese patients with schizophrenia. *Acta Pharmacol Sin,* 33**,** 1409-16.

LIEBERMAN, J., JOHNS, C., POLLACK, S., MASIAR, S., BOOKSTEIN, P., COOPER, T., IADROLA, M., KANE, J., TAMMINGA, C. A. & SCHULZ, S. C. 1991. Biochemical effects of clozapine in cerebrospinal fluid of patients with schizophrenia. New York, NY: Raven Press, Publishers.

LIEBERMAN, J. A., KANE, J. M., SAFFERMAN, A. Z., POLLACK, S., HOWARD, A., SZYMANSKI, S., MASIAR, S. J., KRONIG, M. H., COOPER, T. & NOVACENKO, H. 1994. Predictors of response to clozapine. *J Clin Psychiatry,* 55 Suppl B**,** 126-8.

LINDENMAYER, J.-P. & APERGI, F.-S. 1996. The relationship between clozapine plasma levels and clinical response. *Psychiatric Annals,* 26**,** 406-412.

LIU, H. C., CHANG, W. H., WEI, F. C., LIN, S. K., LIN, S. K. & JANN, M. W. 1996. Monitoring of plasma clozapine levels and its metabolites in refractory schizophrenic patients. *Ther Drug Monit,* 18**,** 200-7.

LIU, Y., LI, H. & YAN, M. 2012. Individual differences in the pharmacokinetics of clozapine in healthy Chinese adults. *Klinik Psikofarmakoloji Bulteni,* 22**,** 17-22.

LLORCA, P. M., LANCON, C., DISDIER, B., FARISSE, J., SAPIN, C. & AUQUIER, P. 2002. Effectiveness of clozapine in neuroleptic-resistant schizophrenia: clinical response and plasma concentrations. *J Psychiatry Neurosci,* 27**,** 30-7.

LOPES, C., COSTA, E., CARMO, A., LOPES, M. J., ROCHA, M. & RODRIGUES, F. 2018. Quantification of clozapine and norclozapine by LC-MS/MS in plasma samples: The importance of CLZ/NCZ ratio. *Clinical Chemistry and Laboratory Medicine,* 56**,** eA9.

LU, M. L., CHEN, T. T., KUO, P. H., HSU, C. C. & CHEN, C. H. 2018. Effects of adjunctive fluvoxamine on metabolic parameters and psychopathology in clozapine-treated patients with schizophrenia: A 12-week, randomized, double-blind, placebo-controlled study. *Schizophr Res,* 193**,** 126-133.

LU, M. L., LANE, H. Y., CHEN, K. P., JANN, M. W., SU, M. H. & CHANG, W. H. 2000. Fluvoxamine reduces the clozapine dosage needed in refractory schizophrenic patients. *J Clin Psychiatry,* 61**,** 594-9.

LUISA IMAZ, M., ORIOLO, G., TORRA, M., SOY, D., GARCIA-ESTEVE, L. & MARTIN-SANTOS, R. 2018. Clozapine use during pregnancy and lactation: A case-series report. *Frontiers in Pharmacology,* 9.

LUSTENBERGER, D. P., GRANATA, J. D. & SCHARSCHMIDT, T. J. 2011. Periostitis secondary to prolonged voriconazole therapy in a lung transplant recipient. *Orthopedics,* 34**,** e793-6.

MA, X., ZHANG, X., GAO, Y., YU, W. & LIU, Q. 2018. Why green tea reduces heart disease risks. *Eur J Prev Cardiol,* 25**,** 1114.

MATSUDA, K. T., CHO, M. C., LIN, K. M., SMITH, M. W., YOUNG, A. S. & ADAMS, J. A. 1996. Clozapine dosage, serum levels, efficacy, and side-effect profiles: a comparison of Korean-American and Caucasian patients. *Psychopharmacol Bull,* 32**,** 253-7.

MAURI, M., VOLONTERI, L. S., FIORENTINI, A., INVERNIZZI, G., NERINI, T., BALDI, M. & BAREGGI, S. R. 2004. Clinical outcome and plasma levels of clozapine and norclozapine in drug-resistant schizophrenic patients. *Schizophr Res,* 66**,** 197-8.

MAURI, M. C., VOLONTERI, L. S., DELL'OSSO, B., REGISPANI, F., PAPA, P., BALDI, M. & BAREGGI, S. R. 2003. Predictors of clinical outcome in schizophrenic patients responding to clozapine. *J Clin Psychopharmacol,* 23**,** 660-4.

MAYEROVA, M., USTOHAL, L., JARKOVSKY, J., PIVNICKA, J., KASPAREK, T. & CESKOVA, E. 2018. Influence of dose, gender, and cigarette smoking on clozapine plasma concentrations. *Neuropsychiatr Dis Treat,* 14**,** 1535-1543.

MCKEAN, A., VELLA-BRINCAT, J. & BEGG, E. 2008. Prescribing and monitoring clozapine in Christchurch. *Australas Psychiatry,* 16**,** 263-7.

MEEHAN, T., WANG, H., DRUMMOND, A. & LOCKMAN, H. 2019. Therapeutic drug monitoring (TDM) during maintenance phase treatment at a community mental health centre. *Australasian Psychiatry,* 27**,** 637-640.

MEIKLEJOHN, H., MOSTAID, M. S., LUZA, S., MANCUSO, S. G., KANG, D., ATHERTON, S., ROTHMOND, D. A., WEICKERT, C. S., OPAZO, C. M., PANTELIS, C., BUSH, A. I., EVERALL, I. P. & BOUSMAN, C. A. 2019. Blood and brain protein levels of ubiquitin-conjugating enzyme E2K (UBE2K) are elevated in individuals with schizophrenia. *J Psychiatr Res,* 113**,** 51-57.

MELENT'EV, A. B. & KUROCHKINA, I. V. 1999. [The determination of clozapine in blood and urine by gas chromatography-mass spectrometry]. *Sud Med Ekspert,* 42**,** 27-9.

MELKERSSON, K. I., HULTING, A.-L. & BRISMAR, K. E. 1999. Different influences of classical antipsychotics and clozapine on glucose–insulin homeostasis in patients with schizophrenia or related psychosis. *The Journal of Clinical Psychiatry,* 60**,** 783-791.

MELKERSSON, K. I. & HULTING, A. L. 2001. Insulin and leptin levels in patients with schizophrenia or related psychoses--a comparison between different antipsychotic agents. *Psychopharmacology (Berl),* 154**,** 205-12.

MELKERSSON, K. I., SCORDO, M. G., GUNES, A. & DAHL, M. L. 2007. Impact of CYP1A2 and CYP2D6 polymorphisms on drug metabolism and on insulin and lipid elevations and insulin resistance in clozapine-treated patients. *J Clin Psychiatry,* 68**,** 697-704.

MELTZER, H. Y. 1992. Treatment of the neuroleptic-nonresponsive schizophrenic patient. *Schizophr Bull,* 18**,** 515-42.

MELTZER, H. Y. 2015. Attention Must Be Paid: The Association of Plasma Clozapine/NDMC Ratio With Working Memory. *Am J Psychiatry,* 172**,** 502-4.

MELTZER, H. Y., BASTANI, B., RAMIREZ, L. & MATSUBARA, S. 1989. Clozapine: new research on efficacy and mechanism of action. *Eur Arch Psychiatry Neurol Sci,* 238**,** 332-9.

MELTZER, H. Y., KENNEDY, J., DAI, J., PARSA, M. & RILEY, D. 1995. Plasma clozapine levels and the treatment of L-DOPA-induced psychosis in Parkinson's disease. A high potency effect of clozapine. *Neuropsychopharmacology,* 12**,** 39-45.

MELZER-RIBEIRO, D. L., RIGONATTI, S. P., KAYO, M., RIBEIRO, R. B., AVRICHIR, B., FORTES, M., CAMARGO, M. E. & ELKIS, H. 2015. Pilot double blind, placebo controlled and randomized study to assess electroconvulsive therapy efficacy as augmenting strategy to clozapine in super-refractory schizophrenia. *Schizophrenia Bulletin,* 1**,** S323-S324.

MENKES, D. B., GLUE, P., GALE, C., LAM, F., HUNG, C. T. & HUNG, N. 2018. Steady-State Clozapine and Norclozapine Pharmacokinetics in Maori and European Patients. *EBioMedicine,* 27**,** 134-137.

MENNICKENT, S., SOBARZO, A., VEGA, M., DE DIEGO, M., GODOY, G., RIOSECO, P. & SAAVEDRA, L. 2010. Determination of clozapine in serum of patients with schizophrenia as a measurement of medication compliance. *Int J Psychiatry Clin Pract,* 14**,** 41-6.

MENNICKENT, S., SOBARZO, A., VEGA, M., GODOY, C. G. & DE DIEGO, M. 2007. Quantitative determination of clozapine in serum by instrumental planar chromatography. *J Sep Sci,* 30**,** 2167-72.

MERLI, R. & HAEFELE, E. 2011. Therapeutic drug monitoring use in clinical practice of a mental health service. *Pharmacopsychiatry. Conference: 9th International Congress of the Interdisciplinary AGNP TDM Task Force. Bolzano Italy. Conference Publication:,* 21.

MEYER, J. M. 2001. Individual changes in clozapine levels after smoking cessation: results and a predictive model. *J Clin Psychopharmacol,* 21**,** 569-74.

MEYER, M. C., BALDESSARINI, R. J., GOFF, D. C. & CENTORRINO, F. 1996. Clinically significant interactions of psychotropic agents with antipsychotic drugs. *Drug Saf,* 15**,** 333-46.

MHRA 2021. MHRA advises monitoring blood concentrations of clozapine and other antipsychotics. *Drug Ther Bull,* 59**,** 37.

MILLER, D. D. 1991. Effect of phenytoin on plasma clozapine concentrations in two patients. *J Clin Psychiatry,* 52**,** 23-5.

MILLER, D. D. 1996. The clinical use of clozapine plasma concentrations in the management of treatment-refractory schizophrenia. *Ann Clin Psychiatry,* 8**,** 99-109.

MILLER, D. D., FLEMING, F., HOLMAN, T. L. & PERRY, P. J. 1994. Plasma clozapine concentrations as a predictor of clinical response: a follow-up study. *J Clin Psychiatry,* 55 Suppl B**,** 117-21.

MOLINS, C., CARCELLER-SINDREU, M., NAVARRO, H., CARMONA, C., PIÑEIRO, M., MARTÍNEZ, E., ÁLVAREZ, E. & PORTELLA, M. J. 2017. Plasma ratio of clozapine to N-desmethylclozapine can predict cognitive performance in treatment-resistant psychotic patients. *Psychiatry Research,* 258**,** 153-157.

MOROZOVA, M., POTANIN, S., BURMINSKIY, D. & BENIASHVILI, A. 2018. Antipsychotic's plasma level variability and its influence on treatment response in schizophrenia exacerbation. *European Psychiatry,* 48**,** S316.

MOUAFFAK, F., TRANULIS, C., GOUREVITCH, R., POIRIER, M. F., DOUKI, S., OLIE, J. P., LOO, H. & GOURION, D. 2006. Augmentation strategies of clozapine with antipsychotics in the treatment of ultraresistant schizophrenia. *Clin Neuropharmacol,* 29**,** 28-33.

MOWERMAN, S. & SIRIS, S. G. 1996. Adjunctive loxapine in a clozapine-resistant cohort of schizophrenic patients. *Ann Clin Psychiatry,* 8**,** 193-7.

MUNRO, J., MATTHIASSON, P., OSBORNE, S., TRAVIS, M., PURCELL, S., COBB, A. M., LAUNER, M., BEER, M. D. & KERWIN, R. 2004. Amisulpride augmentation of clozapine: an open non-randomized study in patients with schizophrenia partially responsive to clozapine. *Acta Psychiatr Scand,* 110**,** 292-8.

NAGENDRAPPA, S., CHHABRA, H., VANTEEMAR, S. S., PARLIKAR, R., DINAKARAN, D., KUMAR, V., RAO, N., KESAVAN, M., VARAMBALLY, S. & VENKATASUBRAMANIAN, G. 2019. "effect of clozapine on frontal activity in treatment resistant schizophrenia-a prospective study using functional near infrared spectroscopy". *Schizophrenia Bulletin,* 45**,** S288-S289.

NG, C. H., CHONG, S. A., LAMBERT, T., FAN, A., HACKETT, L. P., MAHENDRAN, R., SUBRAMANIAM, M. & SCHWEITZER, I. 2005. An inter-ethnic comparison study of clozapine dosage, clinical response and plasma levels. *Int Clin Psychopharmacol,* 20**,** 163-8.

NIJENHUIS, M. & VAN RHENEN, M. 2016. The most prevalent variant allele of CYP1A2 does not affect clozapine and olanzapine pharmacokinetics. *Drug Metabolism and Personalized Therapy,* 31**,** eA58-eA59.

NORMANN, C., HESSLINGER, B., BAUER, J., BERGER, M. & WALDEN, J. 1998. [Significance of hepatic cytochrome P450 enzymes for psychopharmacology]. *Nervenarzt,* 69**,** 944-55.

OLESEN, O. V. 1998. Therapeutic drug monitoring of clozapine treatment. Therapeutic threshold value for serum clozapine concentrations. *Clin Pharmacokinet,* 34**,** 497-502.

OLOYEDE, E., DZAHINI, O., WHISKEY, E. & TAYLOR, D. 2020. Clozapine and Norclozapine Plasma Levels in Patients Switched Between Different Liquid Formulations. *Ther Drug Monit,* 42**,** 491-496.

ORDONEZ, A. E., DRIVER, D. I., ORLOFF, M., PENZANK, S. R., OVERMAN, J., GREENSTEIN, D., DILLARD-BROADNAX, D., RAPOPORT, J. & GOGTAY, N. 2014. The effect of blood plasma clozapine levels on specific measures of behavior in childhood onset schizophrenia. *Schizophrenia Research,* 1**,** S275.

PALEGO, L., BIONDI, L., GIANNACCINI, G., SARNO, N., ELMI, S., CIAPPARELLI, A., CASSANO, G. B., LUCACCHINI, A., MARTINI, C. & DELL'OSSO, L. 2002. Clozapine, norclozapine plasma levels, their sum and ratio in 50 psychotic patients: influence of patient-related variables. *Prog Neuropsychopharmacol Biol Psychiatry,* 26**,** 473-80.

PATTEET, L., MAUDENS, K. E., VERMEULEN, Z., DOCKX, G., DE DONCKER, M., MORRENS, M., SABBE, B. & NEELS, H. 2014. Retrospective evaluation of therapeutic drug monitoring of clozapine and norclozapine in Belgium using a multidrug UHPLC-MS/MS method. *Clin Biochem,* 47**,** 336-9.

PAULS, K. A. M., BROCKELMANN, P. J., HAMMESFAHR, S., BECKER, J., HELLERBACH, A., VISSER-VANDEWALLE, V., DEMBEK, T. A., MEISTER, I. G. & TIMMERMANN, L. 2018. Dysarthria in pallidal Deep Brain Stimulation in dystonia depends on the posterior location of active electrode contacts: a pilot study. *Parkinsonism Relat Disord,* 47**,** 71-75.

PERRY, P. J., BEVER, K. A., ARNDT, S. & COMBS, M. D. 1998. Relationship between patient variables and plasma clozapine concentrations: a dosing nomogram. *Biol Psychiatry,* 44**,** 733-8.

PINZON ESPINOSA, J., GONZALEZ-RODRIGUEZ, A., PENADES, R., SOLERDELCOLL, M., GUTIERREZ, B., PEREZ-BLANCO, F., VAZQUEZ, M., BERNARDO, M., ARRANZ, M. J. & CATALAN, R. 2017. MDR1 and CYP3A4 genes variants association with clinical response measured on a five-factor model in multiepisodic schizophrenia and related disorders. *European Neuropsychopharmacology,* 27**,** S973.

PIWOWARSKA, J., RADZIWON-ZALESKA, M., DMOCHOWSKA, M., SZEPIETOWSKA, E., MATSUMOTO, H., SYGITOWICZ, G., PILC, A. & LUKASZKIEWICZ, J. 2016. The usefulness of monitored therapy using Clozapine concentration in the blood serum for determining drug dose in Polish schizophrenic patients. *Pharmacol Rep,* 68**,** 1120-1125.

POTANIN, S. S., BURMINSKIY, D. S., MOROZOVA, M. A., PLATOVA, A. I., BAYMEEVA, N. V. & MIROSHNICHENKO, II 2015. [Plasma levels of antipsychotics and the severity of side-effects in the treatment of schizophrenia exacerbation]. *Zh Nevrol Psikhiatr Im S S Korsakova,* 115**,** 40-46.

PRÉTERRE, P. 1995. Clozapinémie et ajustement thérapeutique = Clozapine plasma levels and dose adaptation. *L'Encéphale: Revue de psychiatrie clinique biologique et thérapeutique,* 21**,** 53-57.

PRISCO, V., IANNACCONE, T. & FABRAZZO, M. 2015. Nor-clozapine plasma concentration/daily clozapine dose ratio: An index of response to clozapine treatment. *European Neuropsychopharmacology,* 2**,** S484-S485.

QIU, X. W., HE, X. J., HUANG, S. H., LIANG, B. B. & YU, Z. R. 2015. [New progress on three-dimensional movement measurement analysis of human spine]. *Zhongguo Gu Shang,* 28**,** 476-81.

RAASKA, K. & NEUVONEN, P. J. 2000. Ciprofloxacin increases serum clozapine and N-desmethylclozapine: a study in patients with schizophrenia. *Eur J Clin Pharmacol,* 56**,** 585-9.

RAJKUMAR, A. P., CHITRA, C., BHUVANESHWARI, S., POONKUZHALI, B., KURUVILLA, A. & JACOB, K. S. 2011. Clinical Predictors of Response to Clozapine in Patients with Treatment Resistant Schizophrenia. *Psychopharmacol Bull,* 44**,** 51-65.

RAJKUMAR, A. P., POONKUZHALI, B., KURUVILLA, A., JACOB, M. & JACOB, K. S. 2013. Clinical predictors of serum clozapine levels in patients with treatment-resistant schizophrenia. *Int Clin Psychopharmacol,* 28**,** 50-6.

RENNE, D., BURGY, M., WIEL, , E. & SHIPKOVA, M. 2016. Application of the AGNP consensus guidelines for therapeutic drug monitoring in psychiatry to patients under therapy with the antipsychotics clozapine, olanzapine, and risperidone in a community hospital in Germany. *Pharmacopsychiatry. Conference: 12th Symposium of the AGNP Task Force on Therapeutic Drug Monitoring in Psychiatry. Germany.,* 26.

REPO-TIIHONEN, E., ELORANTA, A., HALLIKAINEN, T. & TIIHONEN, J. 2005. Effects of venlafaxine treatment on clozapine plasma levels in schizophrenic patients. *Neuropsychobiology,* 51**,** 173-6.

ROSTAMI-HODJEGAN, A., AMIN, A. M., SPENCER, E. P., LENNARD, M. S., TUCKER, G. T. & FLANAGAN, R. J. 2004. Influence of dose, cigarette smoking, age, sex, and metabolic activity on plasma clozapine concentrations: a predictive model and nomograms to aid clozapine dose adjustment and to assess compliance in individual patients. *J Clin Psychopharmacol,* 24**,** 70-8.

SAITO, Y., IIZUKA, T., KORETSUNE, T., ARITA, R., SHIMIZU, S. & IWASA, Y. 2016. Gate-Tuned Thermoelectric Power in Black Phosphorus. *Nano Lett,* 16**,** 4819-24.

SALAZAR-PEREYRA, A., TOME, I. S. R., RAMIREZ-BERMUDEZ, J., CASTRO-ROMAN, R., CASTRO-TORRES, N. N. & JUNG-COOK, H. 2011. Monitoring of plasma clozapine concentrations in patients with schizophrenia. [Spanish]. *Archivos de Neurociencias,* 16**,** 4-7.

SAMANAITE, R., GILLESPIE, A., SENDT, K. V., MCQUEEN, G., MACCABE, J. H. & EGERTON, A. 2018. Biological Predictors of Clozapine Response: A Systematic Review. *Front Psychiatry,* 9**,** 327.

SCHIRMBECK, F. & ZINK, M. 2013. Comorbid obsessive-compulsive symptoms in schizophrenia: contributions of pharmacological and genetic factors. *Front Pharmacol,* 4**,** 99.

SCHORETSANITIS, G., KANE, J. M., RUAN, C. J., SPINA, E., HIEMKE, C. & DE LEON, J. 2019. A comprehensive review of the clinical utility of and a combined analysis of the clozapine/norclozapine ratio in therapeutic drug monitoring for adult patients. *Expert Rev Clin Pharmacol,* 12**,** 603-621.

SCHORETSANITIS, G., MENDELOWITZ, A., MALUR, C., BRAGA, R. J., SCHOOLER, N. R., JOHN, M., DE LEON, J., KANE, J. M. & PETRIDES, G. 2020. Lack of ECT effects on clozapine plasma levels in patients with treatment-resistant schizophrenia: Pharmacokinetic evidence from a randomized clinical trial. *Schizophr Res,* 218**,** 309-311.

SCHORETSANITIS, G., SMITH, R. L., MOLDEN, E., SOLISMAA, A., SEPPALA, N., KOPECEK, M., SVANCER, P., OLMOS, I., VAZQUEZ, M., IGLESIAS-GARCIA, C., IGLESIAS-ALONSO, A., SPINA, E. & DE LEON, J. 2021. European Whites May Need Lower Minimum Therapeutic Clozapine Doses Than Those Customarily Proposed. *J Clin Psychopharmacol,* 41**,** 140-147.

SCHULTE, P. F. J. 2003. Clozapine in treatment-refractory schizophrenia - Plasma level and duration of an adequate treatment trial. [German]. *Psychopharmakotherapie,* 10**,** 102-111.

SCHULZ, E., FLEISCHHAKER, C. & REMSCHMIDT, H. 1995. Determination of clozapine and its major metabolites in serum samples of adolescent schizophrenic patients by high-performance liquid chromatography. Data from a prospective clinical trial. *Pharmacopsychiatry,* 28**,** 20-5.

SEPPALA, N. H., LEINONEN, E. V., LEHTONEN, M. L. & KIVISTO, K. T. 1999. Clozapine serum concentrations are lower in smoking than in non-smoking schizophrenic patients. *Pharmacol Toxicol,* 85**,** 244-6.

SHANG, D. W., LI, L. J., WANG, X. P., WEN, Y. G., REN, Y. P., GUO, W., LI, W. B., LI, L., ZHOU, T. Y., LU, W. & WANG, C. Y. 2014. Population pharmacokinetic/pharmacodynamic model of clozapine for characterizing the relationship between accumulated exposure and PANSS scores in patients with schizophrenia. *Ther Drug Monit,* 36**,** 378-86.

SINGH, H., DUBIN, W. R. & KAUR, S. 2015. Drug interactions affecting clozapine levels. *Journal of Psychiatric Intensive Care,* 11**,** 52-65.

SPINA, E., AVENOSO, A., FACCIOLA, G., FABRAZZO, M., MONTELEONE, P., MAJ, M., PERUCCA, E. & CAPUTI, A. P. 1998. Effect of fluoxetine on the plasma concentrations of clozapine and its major metabolites in patients with schizophrenia. *Int Clin Psychopharmacol,* 13**,** 141-5.

SPORN, A. L., VERMANI, A., GREENSTEIN, D. K., BOBB, A. J., SPENCER, E. P., CLASEN, L. S., TOSSELL, J. W., STAYER, C. C., GOCHMAN, P. A., LENANE, M. C., RAPOPORT, J. L. & GOGTAY, N. 2007. Clozapine treatment of childhood-onset schizophrenia: evaluation of effectiveness, adverse effects, and long-term outcome. *J Am Acad Child Adolesc Psychiatry,* 46**,** 1349-1356.

STAHEL, P., CANT, J. P., MACPHERSON, J. A., BERENDS, H. & STEELE, M. A. 2016. A Mechanistic Model of Intermittent Gastric Emptying and Glucose-Insulin Dynamics following a Meal Containing Milk Components. *PLoS One,* 11**,** e0156443.

STARK, A. & SCOTT, J. 2012. A review of the use of clozapine levels to guide treatment and determine cause of death. *Aust N Z J Psychiatry,* 46**,** 816-25.

STIEFFENHOFER, V., SAGLAM, H., SCHMIDTMANN, I., SILVER, H., HIEMKE, C. & KONRAD, A. 2011a. Clozapine plasma level monitoring for prediction of rehospitalization schizophrenic outpatients. *Pharmacopsychiatry,* 44**,** 55-9.

STIEFFENHOFER, V., SAGLAM, H., SCHMIDTMANN, I., SILVER, H., KONRAD, A. & HIEMKE, C. 2011b. Clozapine plasma level monitoring for prediction of relapse in schizophrenic outpatients. *European Archives of Psychiatry and Clinical Neuroscience,* 1**,** S69.

SUBRAMANIAM, M., NG, C., CHONG, S. A., MAHENDRAN, R., LAMBERT, T., PEK, E. & HUAK, C. Y. 2007. Metabolic differences between Asian and Caucasian patients on clozapine treatment. *Hum Psychopharmacol,* 22**,** 217-22.

SUHAS, S., KUMAR, V., DAMODHARAN, D., SHARMA, P., RAO, N. P., VARAMBALLY, S., VENKATASUBRAMANIAN, G., MURTHY, P. & GANGADHAR, B. N. 2020. Do Indian patients with schizophrenia need half the recommended clozapine dose to achieve therapeutic serum level? An exploratory study. *Schizophr Res,* 222**,** 195-201.

SUZUKI, T., UCHIDA, H., WATANABE, K. & KASHIMA, H. 2011. Factors associated with response to clozapine in schizophrenia: a review. *Psychopharmacol Bull,* 44**,** 32-60.

TANG, Y. L., MAO, P., LI, F. M., LI, W., CHEN, Q., JIANG, F., CAI, Z. J. & MITCHELL, P. B. 2007. Gender, age, smoking behaviour and plasma clozapine concentrations in 193 Chinese inpatients with schizophrenia. *Br J Clin Pharmacol,* 64**,** 49-56.

TAYLOR, D., VARMA, S., BISHARA, D. & BESAG, F. M. C. 2011. Clozapine-related EEG changes and seizures: Dose and plasma-level relationships. *Therapeutic Advances in Psychopharmacology,* 1**,** 47-66.

THORNTON, A. E., PROCYSHYN, R. M., BARR, A. M., MACEWAN, G. W. & HONER, W. G. 2015. Cognition and Plasma Ratio of Clozapine to N-desmethylclozapine in Patients With Clozapine-Resistant Schizophrenia. *Am J Psychiatry,* 172**,** 1259.

THORUP, M. & FOG, R. 1977. Clozapine treatment of schizophrenic patients. Plasma concentration and coagulation factors. *Acta Psychiatr Scand,* 55**,** 123-6.

TSUDA, Y., SARUWATARI, J. & YASUI-FURUKORI, N. 2014. Meta-analysis: The effects of smoking on the disposition of two commonly used antipsychotic agents, olanzapine and clozapine. *BMJ Open,* 4.

TURJAP, M. & JURICA, J. 2013. Therapeutic drug monitoring of clozapine and olanzapine. [Czech]. *Psychiatrie,* 17**,** 80-85.

ULRICH, S., BAUMANN, B., WOLF, R., LEHMANN, D., PETERS, B., BOGERTS, B. & MEYER, F. P. 2003. Therapeutic drug monitoring of clozapine and relapse--a retrospective study of routine clinical data. *Int J Clin Pharmacol Ther,* 41**,** 3-13.

VAILLEAU, J. L., JEANNY, B., CHOMARD, P. & VINCENT, R. 1996. [Importance of determining clozapine plasma level in follow-up of schizophrenic patients]. *Encephale,* 22**,** 103-9.

VALLETTE, N., GOSSELIN, O. & KAHN, J. P. 2001. [Efficacy of clozapine in the course of Huntington chorea: apropos of a clinical case]. *Encephale,* 27**,** 169-71.

VAN DER WEIDE, J., STEIJNS, L. S. & VAN WEELDEN, M. J. 2003. The effect of smoking and cytochrome P450 CYP1A2 genetic polymorphism on clozapine clearance and dose requirement. *Pharmacogenetics,* 13**,** 169-72.

VANDERZWAAG, C., MCGEE, M., MCEVOY, J. P., FREUDENREICH, O., WILSON, W. H. & COOPER, T. B. 1996. Response of patients with treatment-refractory schizophrenia to clozapine within three serum level ranges. *Am J Psychiatry,* 153**,** 1579-84.

VERMA, M., GROVER, S. & CHAKRABARTI, S. 2021. Effectiveness of clozapine on quality of life and functioning in patients with treatment-resistant schizophrenia. *Nordic Journal of Psychiatry,* 75**,** 135-144.

VINCENT, P. & HALME, A. 2011. Comparison of hospitalisation time of schizophrenic patients exposed and not exposed to therapeutic drug monitoring of clozapine. *Journal of Pharmacy Practice,* 24**,** 252.

WANG, C. & LI, L. 2012. Proper use of clozapine: Experiences in China. *Shanghai Archives of Psychiatry,* 24**,** 108-109.

WEHRING, H. J., ELSOBKY, T., MCEVOY, J. P., VYAS, G., RICHARDSON, C. M., MCMAHON, R. P., DIPAULA, B. A., LIU, F., SULLIVAN, K., BUCHANAN, R. W., FELDMAN, S., MCMAHON, E. M. & KELLY, D. L. 2018. Adjunctive Minocycline in Clozapine-Treated Patients with Schizophrenia: Analyzing the Effects of Minocycline on Clozapine Plasma Levels. *Psychiatr Q,* 89**,** 73-80.

WENZEL-SEIFERT, K., KOESTLBACHER, A. & HAEN, E. 2011. Dose-dependent effects of cigarette smoking on serum concentrations of psychotropic drugs. *Therapeutic Drug Monitoring,* 33**,** 481-482.

WILBORN, C., PERRY, P., POPISH, S. & CHOU, T. 2011. Assessment of prophylactic clozapine levels in stable outpatients with schizophrenia and schizoaffective disorder. *Journal of Pharmacy Practice,* 24**,** 265.

WOHKITTEL, C., GERLACH, M., TAURINES, R., WEWETZER, C., UNTERECKER, S., BURGER, R., SCHRECK, D., MEHLER-WEX, C., ROMANOS, M. & EGBERTS, K. 2016. Relationship between clozapine dose, serum concentration, and clinical outcome in children and adolescents in clinical practice. *J Neural Transm (Vienna),* 123**,** 1021-31.

WOLDEYOHANNES, D., ASMAMAW, Y., SISAY, S., HAILESSELASSIE, W., BIRMETA, K. & TEKESTE, Z. 2017. Risky HIV sexual behavior and utilization of voluntary counseling and HIV testing and associated factors among undergraduate students in Addis Ababa, Ethiopia. *BMC Public Health,* 17**,** 121.

WONG, J. O., LEUNG, S. P., MAK, T., NG, R. M., CHAN, K. T., HON-KEE CHEUNG, H., CHOI, W. K., LAI, J. & WAI-KIU TSANG, A. 2006. Plasma clozapine levels and clinical response in treatment-refractory Chinese schizophrenic patients. *Prog Neuropsychopharmacol Biol Psychiatry,* 30**,** 251-64.

XIANG, Y. Q., ZHANG, Z. J., WENG, Y. Z., ZHAI, Y. M., LI, W. B., CAI, Z. J., TAN, Q. R. & WANG, C. Y. 2006. Serum concentrations of clozapine and norclozapine in the prediction of relapse of patients with schizophrenia. *Schizophr Res,* 83**,** 201-10.

YADA, Y., KITAGAWA, K., SAKAMOTO, S., OZAWA, A., NAKADA, A., KASHIWAGI, H., OKAHISA, Y., TAKAO, S., TAKAKI, M., KISHI, Y. & YAMADA, N. 2021. The relationship between plasma clozapine concentration and clinical outcome: a cross-sectional study. *Acta Psychiatr Scand,* 143**,** 227-237.

YAĞCIOĞLU, A. E. A., AKDEDE, B. B. K., TURGUT, T. İ., TÜMÜKLÜ, M., YAZICI, M. K., ALPTEKIN, K., ERTUĞRUL, A., JAYATHILAKE, K., GÖĞÜŞ, A., TUNCA, Z. & MELTZER, H. Y. 2005. A Double-Blind Controlled Study of Adjunctive Treatment With Risperidone in Schizophrenic Patients Partially Responsive to Clozapine: Efficacy and Safety. *The Journal of Clinical Psychiatry,* 66**,** 63-72.

YAMAGUCHI, K., FERIL, L. B., JR., HARADA, Y., ENDO, H., IRIE, Y., NAKAYAMA, J. & TACHIBANA, K. 2009. Growth inhibition of neurofibroma by ultrasound-mediated interferon gamma transfection. *J Med Ultrason (2001),* 36**,** 3-8.

YELMO-CRUZ, S., PAICO-RODRIGUEZ, D. F., DIAZ-MESA, E., VERA-BARRIOS, E., MORERA-FUMERO, A. L., ABREU-GONZALEZ, P., HENRY-BENITEZ, M., CEJAS-MENDEZ, M. R. & GRACIA-MARCO, R. 2016. Can we maintain plasma clozapine levels above the upper limit? *European Neuropsychopharmacology,* 26**,** S559.

YOUN, T., CHUNG, I. W., KIM, H. S., KIM, S. H., LEE, N. Y. & KIM, Y. S. 2016. Electroconvulsive therapy augmentation in clozapine-resistant schizophrenia. *European Neuropsychopharmacology,* 26**,** S530.

YUAN, H. Y., LI, H. D. & WANG, L. H. 2002. The application of decision analysis to the TDM of clozapine. [Chinese]. *Chinese Pharmaceutical Journal,* 37**,** 684-686.

YUANGUANG, C., JINGPING, Z. & GUANGRONG, X. 1998. A study on serum concentration and clinical response to clozapine with different dose administration for treatment of schizophrenia. *Chin J Psychiatry,* 31**,** 104-107.

YUE, Y., XU, Y. F., SONG, L. S., YI, Z. H. & LU, W. H. 2005. Association of smoking with the therapeutic effects of the drug, side effects and plasma level of drug in patients with schizophrenia. [Chinese]. *Chinese Journal of Clinical Rehabilitation,* 9**,** 44-45.

ZAI, G. C., ZAI, C. C., CHOWDHURY, N. I., TIWARI, A. K., SOUZA, R. P., LIEBERMAN, J. A., MELTZER, H. Y., POTKIN, S. G., MULLER, D. J. & KENNEDY, J. L. 2012. The role of brain-derived neurotrophic factor (BDNF) gene variants in antipsychotic response and antipsychotic-induced weight gain. *Prog Neuropsychopharmacol Biol Psychiatry,* 39**,** 96-101.

ZHOU, S. F., YANG, L. P., ZHOU, Z. W., LIU, Y. H. & CHAN, E. 2009. Insights into the substrate specificity, inhibitors, regulation, and polymorphisms and the clinical impact of human cytochrome P450 1A2. *AAPS J,* 11**,** 481-94.

ZIVKOVIC, N., PAVICEVIC, D., DJOKIC, G. & LEKOVIC, J. 2009. Effect of sigarette smoking on treatment of schizophrenia with clozapine. *European Neuropsychopharmacology,* 3**,** S670-S671.
